# Molecular cannibalism: Sacrificial materials as precursors for hollow and multidomain single crystals

**DOI:** 10.1038/s41467-021-21076-9

**Published:** 2021-02-11

**Authors:** Maria Chiara di Gregorio, Merna Elsousou, Qiang Wen, Linda J. W. Shimon, Vlad Brumfeld, Lothar Houben, Michal Lahav, Milko E. van der Boom

**Affiliations:** 1 Department of Organic Chemistry, Weizmann Institute of Science, Rehovot, Israel; 2 Department of Chemical Research Support, Weizmann Institute of Science, Rehovot, Israel

**Keywords:** Crystal engineering, Molecular self-assembly, Organic molecules in materials science

## Abstract

The coexistence of single-crystallinity with a multidomain morphology is a paradoxical phenomenon occurring in biomineralization. Translating such feature to synthetic materials is a highly challenging process in crystal engineering. We demonstrate the formation of metallo-organic single-crystals with a unique appearance: six-connected half-rods forming a hexagonal-like tube. These uniform objects are formed from unstable, monodomain crystals. The monodomain crystals dissolve from the inner regions, while material is anisotropically added to their shell, resulting in hollow, single-crystals. Regardless of the different morphologies and growth mechanism, the crystallographic structures of the mono- and multidomain crystals are nearly identical. The chiral crystals are formed from achiral components, and belong to a rare space group (*P*622). Sonication of the solvents generating radical species is essential for forming the multidomain single-crystals. This process reduces the concentration of the active metal salt. Our approach offers opportunities to generate a new class of crystals.

## Abstract (web summary)

The coexistence of single-crystallinity with a multidomain morphology is a paradoxical crystallographic phenomenon. Here, the authors introduce a crystallographic morphology never reported before. The single-crystals with a curved and hollow morphology offer opportunities to generate a class of synthetic multidomain crystals.

## Introduction

The properties and functionalities of crystals are strongly affected by their shape and structure. The shape-based relationship is manifested in both biological and synthetic systems. Single crystalline scaffolds having hierarchical architectures and curved features are widely used for structural purposes by many plants and animals (e.g., mollusks, corals, echinoderms, and algae)^1,2^. Sea urchins sculpt their spines by producing single crystals of calcite having a complex fenestrated morphology with smooth and curved surfaces^3^. Among synthetic crystals, the shape of nanoparticles has been shown to affect optical properties^4,5^ and impact mechanical properties as well as cell membrane permeability^6^. The latter property is important for their efficacy for drug delivery and other therapeutic applications^6,7^. However, unlike bio-crystallization, where crystal shaping is under cellular control, mastering of crystallization chemistry and kinetics is required for synthetic materials. Various parameters such as solvents and additives can control the evolution of crystal facets by selective surface interactions^8–10^. Interfacial synthesis^11^, microemulsion^12^, and template-assisted growth^13^ are transversally used in colloidal chemistry to enhance the performance of both organic^14,15^ and inorganic materials^16,17^ such as drugs and functional nanoparticles^18,19^.

The synthesis of morphologically tailored metal-organic frameworks (MOFs) is still in its early stages^20^. Although several works have shown that control over crystal size and shape can affect porosity^21^, catalytic activity^22^, and cellular uptake^23^, to date, the majority of the efforts have been aimed at designing the crystal structures of MOFs^24^. This fact has confined much of the ongoing research to applications that exploit the porosity of the molecular frameworks^25,26^. Such crystals have rarely been utilized as three dimensional objects^27^. The shaping processes are mainly restricted to crystals with canonical frameworks and their fundamental aspects are poorly understood^20^. Our group has recently shown that a variability of uniform metallo-organic crystals with different morphologies can be obtained^28–30^. These studies include the formation of a unique *yoyo*-shaped, single crystal exhibiting both a multidomain and chiral morphology^30^. The coexistence of such a set of properties broke the axiom “morphological single unit–monocrystallinity” by reproducing a phenomenon previously observed in bio-minerals^31,32^.

**Fig. 1:**
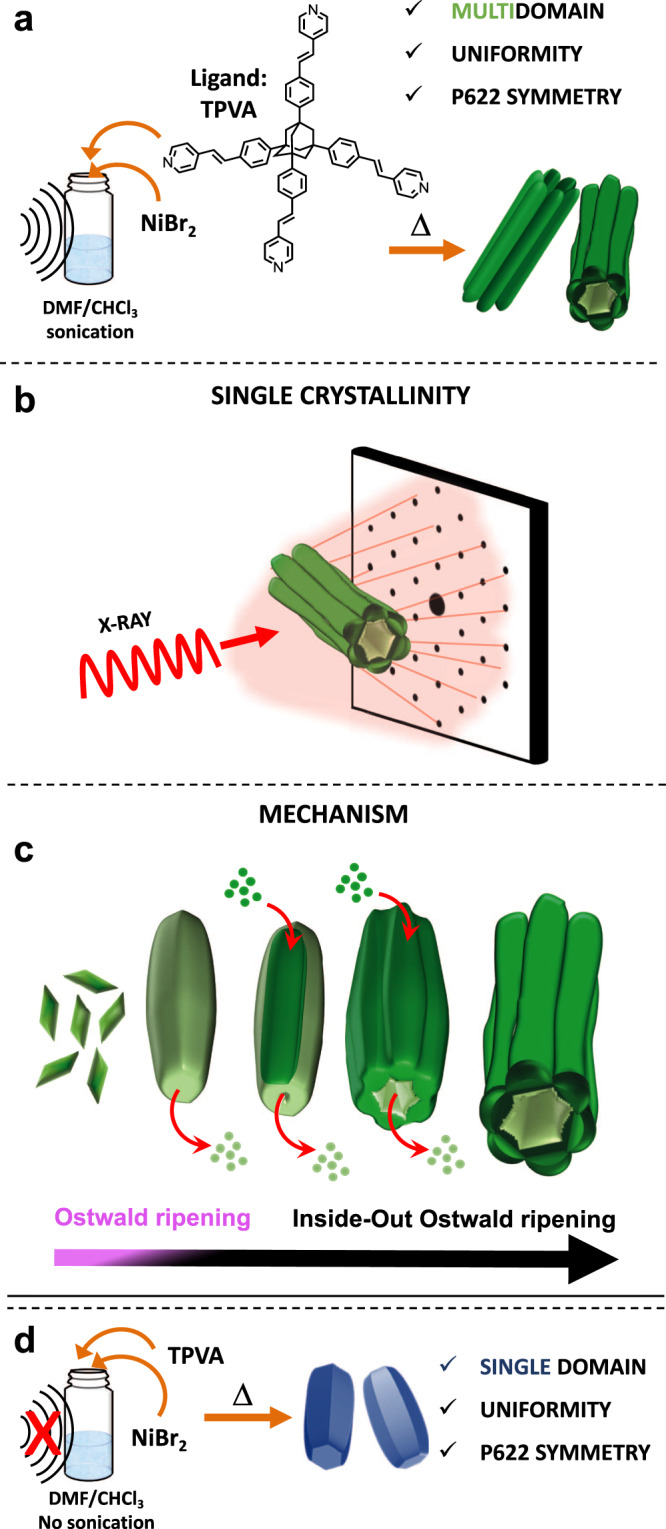
Single, uniform, and multidomain-shaped crystals: a hollow, metal-organic framework (MOF) formed under sonochemical–solvothermal conditions. **a** Crystals consisting of six protruding units were obtained after 48 h by reacting the organic ligand (TPVA) and nickel(II) bromide at 105 °C in a mixture of DMF and CHCl_3_ (3:1 v/v). The solvents were first sonicated for 1.5 h prior to the addition of TPVA and the metal salt (TPVA:NiBr_2_ = 1:2). **b** Drawing of a X-ray diffraction pattern typical for single crystals. **c** Inside-out Ostwald ripening, resulting in large continuous channels and a multidomain morphology. **d** Isostructural crystals having a prismatic single domain (monodomain) morphology were formed without sonication.

In this work, we introduce an unknown crystal shape (Fig. 1). The entire crystals are hollow and have a multidomain appearance. Their single-crystallinity and chiral packing is evident from detailed crystallographic studies. A straightforward but unconventionally additive-free synthesis is described here, where the key step is the sonication of the reaction solvents to reduce the active concentration of the metal salt. Incipient monodomain, single crystals are sacrificial templates for the formation of the thermodynamically favored multidomain crystals; their instability results from internal structural defects generated by speed-up growth kinetics. The multidomain crystals have curved morphological features and retain single-crystallinity.

## Results

### Formation of hollow crystals with a multidomain morphology

The tetrahydral organic ligand (TPVA) with nickel bromide (NiBr_2_) underwent a sonochemical–solvothermal treatment in a 1:2 molar ratio to form the hollow crystals. A mixture of dimethylformamide (DMF)/chloroform (v/v = 2:1) was sonicated for 1.5 h before the TPVA was added. Then, the solution containing the TPVA was mixed with a DMF solution of the metal salt in a glass pressure tube and heated at 105 °C for 48 h, resulting in the formation of a green precipitate (Fig. 1a). An excess of the metal salt was used: (1) to ensure the formation of a fully coordinative network (four pyridine units of four TPVA ligands are coordinated to one metal center, vide infra), and (2) to enhance morphological uniformity by surface saturation^28^. Sonication is known to result in the formation of radicals^33,34^, which affects crystal growth^35^. Indeed, electron paramagnetic resonance (EPR) measurements indicated the formation of radicals (vide infra, Supplementary Fig. 1) having a lifetime of ~45 min at room temperature, in the presence of the metal salt.

**Fig. 2:**
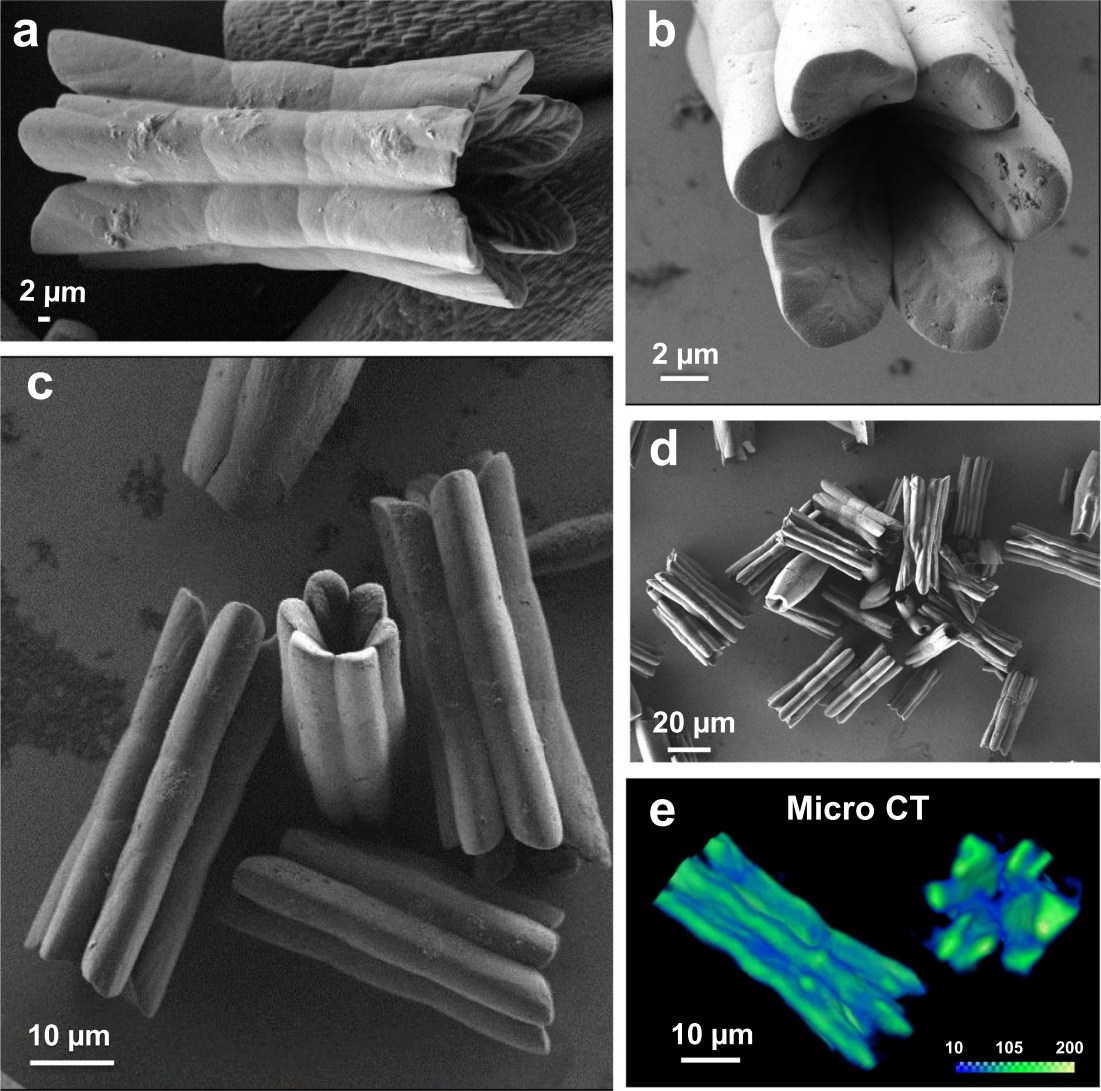
Hollow crystals with a multidomain appearance. **a**–**d** Scanning electron microscopy (SEM) images and **e** micro-computed tomography (Micro-CT) volume rendering of the crystals (sonochemical–solvothermal conditions, 105 °C, *t* = 48 h). The color legend in **e** shows the Hounsfield units values, which are proportional to the amount of material.

**Fig. 3:**
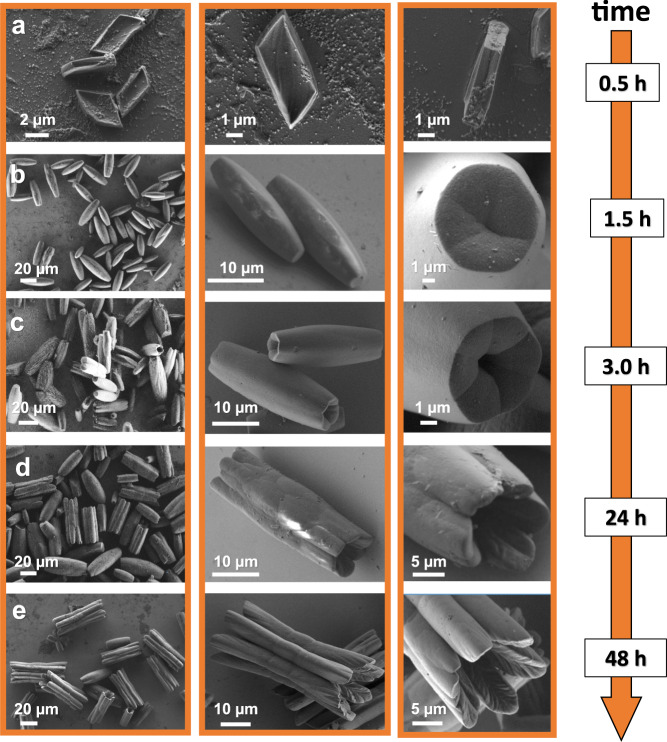
Evolution of morphological complexity. **a**–**e **A series of ex situ scanning electron microscopy (SEM) images showing the stages in the temporal evolution of MOF-NiBr_2_ (sonochemical–solvothermal conditions, 105 °C). Left: zoom-out view and zoom-in micrographs (central, right).

**Fig. 4:**
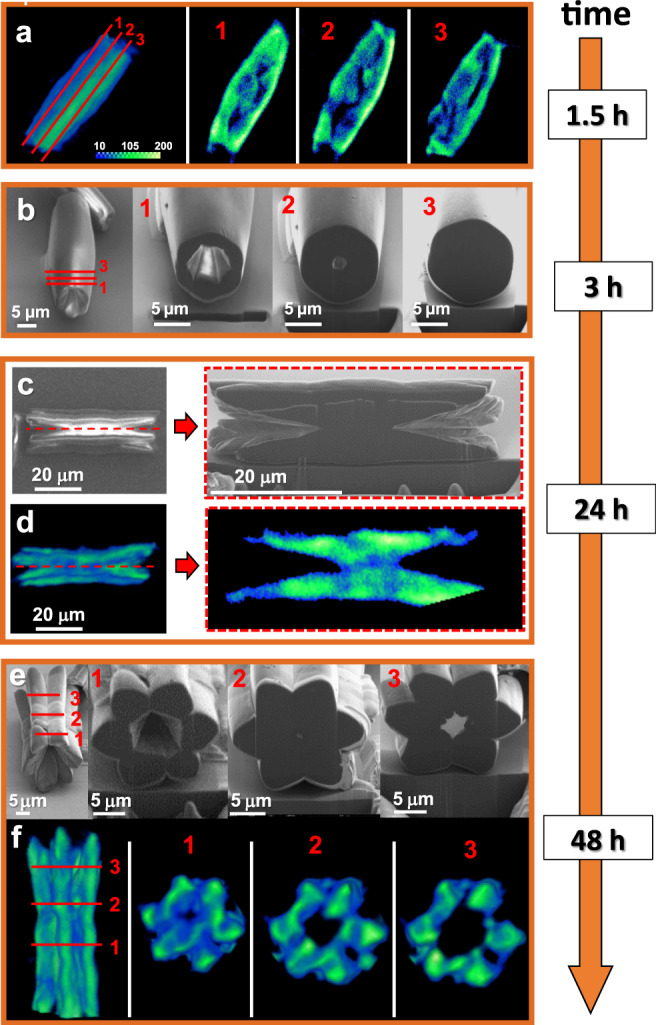
Evolution of the continuous, double-cone-shaped channels. Series of ex situ micro-computed tomography (Micro-CT) volume renderings (**a**, **d**, **f**) and scanning electron microscopy (SEM) images (**b**, **c**, **e**) showing snapshots of the formation of MOF-NiBr_2_ with time (sonochemical–solvothermal conditions, 105 °C). The cross-sections shown in **b**, **c**, **e** obtained with the focused ion beam (FIB) microscope are denoted by red lines and are numbered.

SEM images of the precipitate show the formation of hollow, multidomain structures (Fig. 2a–d and Supplementary Fig. 2). These crystals consist of six-connected half-rods that are positioned circularly. Regardless of their complex and highly unusual morphology, their uniformity is high (*l* = 35.7 ± 5.1 μm, *⌀*_*out*_ = 13.6 ± 2.3 μm). Morphologically, these half-rods are thinner at their terminal rounded edges and their external surfaces seem to exhibit three structurally similar subdomains. High-resolution SEM images of these rounded termini show large, microsized cavities having a fine-textured, inner surface (Figs. 2b and 3). The textures resemble the main veins of leaves. The entire structures are hollow, as proven by full volume reconstruction and cross-section analysis of micro-computed tomography (micro-CT) data (Figs. 2e and 4 and Supplementary Movie 1). The continuous channel is double-cone shaped; the diameter at the edges is substantially larger (~10 times) than that at the core. Relatively low electron-dense regions are present at the interfaces that connect the half-rods.

### Single-crystallinity and its retention

**Fig. 5:**
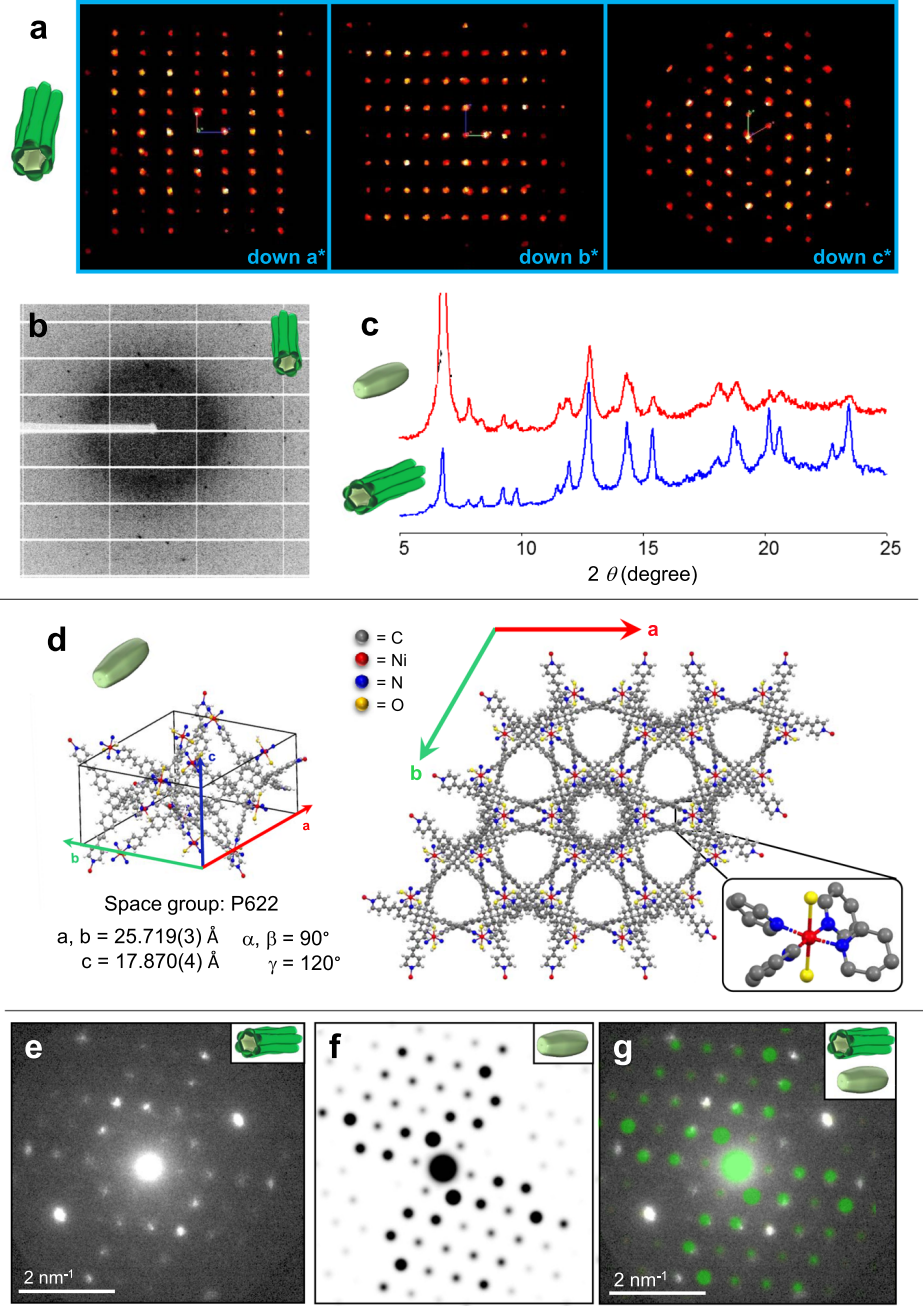
Single-crystallinity coexisting with multidomain morphology. The data shown are for crystals obtained by sonochemical–solvothermal conditions (105 °C). **a** Ewald sphere projections of MOF-NiBr_2_ (*t* = 48 h) down the a*, b*, and c* axes, **b** X-ray experimental diffraction 1 degree frame of the entire crystal of MOF-NiBr_2_ (*t* = 48 h). **c** Powder X-ray diffraction (PXRD) of MOF-NiBr_2_, *t* = 1.5 h (red line) and *t* = 48 h (blue line). **d** Single-crystal X-ray structure of MOF-NiBr_2_ (*t* = 1.5 h). **e** Nanobeam electron diffraction of MOF-NiBr_2_ (*t* = 48 h). **f** Kinematical zone axis diffraction patterns calculated from the single-crystal X-ray data shown in **d** of MOF-NiBr_2_ (*t* = 1.5 h), using electron scattering factors. **g** Comparison between the diffraction data shown in **e** and **f**. Deviating intensities are related to dynamical scattering into reflections that are a sum of reflections in the same zone axis pattern (Supplementary Fig. 16d).

For diffraction analysis, isolated, entire crystals (sonochemical–solvothermal conditions, *t* = 48 h) were subjected to a 100 μm X-ray beam of a home-source diffractometer. This relatively large beam exposes the entire crystal. The observed diffraction patterns were characteristic of a typical single crystal, with well-defined lattice spacings as can be seen from the Ewald’s sphere projection (Fig. 5a). X-ray diffraction data were also obtained from synchrotron radiation (Fig. 5b), albeit, with a 30 μm beam. Again, the reflections could be indexed by a single domain orientation. These measurements did not indicate multiplicity or twinning. The single-crystallinity was consistent with diffraction patterns obtained from transmission electron microscopy (TEM). These crystals and initial crystals isolated after 1.5 h reaction time were analyzed by both powder X-ray diffraction (PXRD) (Fig. 5c and Supplementary Figs. 3a and 4) and by single crystal X-ray diffraction for structural determination at the molecular level (Fig. 5d, Supplementary Figs. 5 and 6, and Supplementary Table 1). The PXRD patterns of both crystals (*t* = 1.5 h and *t* = 48 h) are very similar, indicating that the final crystallographic structure is already established after a relatively short reaction time and is retained during the growth process (Fig. 5c, Supplementary Figs. 3a and 4b, c, and Supplementary Note 1). The diffraction collected from these entire crystals (obtained after 1.5 h) was also typical for single-crystallinity. Moreover, the crystallographic isostructurality is evident by the superposition of the TEM nanobeam electron diffraction pattern (*t* = 48 h) and the single-crystal diffraction pattern in the corresponding viewing direction based on the X-ray structure (*t* = 1.5 h) (Fig. 5e–g). Both crystals are isomorphous and their structures were solved by single-crystal X-ray crystallography (hexagonal space group, *P*622 with unit cell parameters *t* = 1.5 h: *a* = *b* = 25.719 Å, *c* = 17.870 Å versus *t* = 48 h: *a* = *b* = 25.961 Å, *c* = 17.818 Å). The uncommon space group, *P*622, is one of the 65 Sohncke groups; it indicates that the molecular components have chiral packing (Supplementary Table 1). The crystal structures were solved and refined to an atomic resolution of 1.19 Å (*t* = 1.5 h) and 1.10 Å (*t* = 48 h) with a final *R* factor of 0.0762 (*t* = 1.5 h) and 0.0941 (*t* = 48 h) for [*I* > 2σ(*I*)]. The Flack parameters are similar 0.11(9) (*t* = 1.5 h) and 0.13(8) (*t* = 48 h), and indicate that a small amount of enantiomeric twinning could be present in this crystal. Circular dichroism measurements of dispersions of these two crystals in ethanol show a zero signal, indicating there is no enantiomeric excess in these bulk samples (Supplementary Fig. 7). Apparently, the bulk samples consist of a racemic mixture. The achiral components (TPVA and the nickel bromide) are arranged in a complex, continuous network consisting of different helicoids forming supramolecular homochiral channels (Supplementary Figs. 5 and 6). The diameters of these hexagonal (*⌀* ≈ 9.1 Å) and trigonal (*⌀* ≈ 11.6 Å) channels can be observed along the *c*-axis. The bivalent nickel centers have an octahedral geometry, with four pyridine moieties of four TPVA ligands in the equatorial positions (*t* = 1.5 h: Ni–N = 2.021(15) Å and 2.037(14) Å versus *t* = 48 h: Ni–N = 2.100(8) Å and 2.08(11) Å). Two water molecules are bound with their oxygen atoms in the axial positions (*t* = 1.5 h: Ni–O = 2.451(0.007) Å versus *t* = 48 h: Ni–O = 2.427(0.006)). The Ni–N bond distances and lengths are within the range commonly found for coordinately saturated nickel complexes. However, the axial position is longer than usual (the Ni–O distances for analog pyridine-based complexes is ~2.08 Å)^36^. The four pyridine moieties around the metal center are arranged in a chiral, propeller-type conformation, which is most likely the origin of the chirality (Supplementary Fig. 8). The unit cell contain six of these propeller arrangements with the same handedness.

Elemental analyses were performed on the crystals showing that these materials have a similar composition (Supplementary Table 2; sonochemical–solvothermal and solvothermal conditions). Only traces (0.32–0.65%) of bromide (Br^−^) have been observed. The positive charges of the metal centers are balanced by chloride (Cl^−^), as indicated by an average ratio of Cl/Ni = 2.2. These anions (from chloroform)^29,30,34,37–39^ are not resolved as part of the crystallographic structures. The reaction with NiCl_2_ and TPVA under solvothermal conditions resulted in uniform, sub-micron-sized crystals having a different morphology than observed in this study with NiBr_2_^39^. This observation indicate that the bromide–chloride anion exchange happened after coordination of the TPVA to the NiBr_2_.

### From mono- to multidomain single crystals

In order to follow the morphological transformation, a series of ex situ scanning electron microscopy (SEM) measurements and micro-CT measurements were carried out by stopping the solvothermal reaction at different reaction times (Figs. 3 and 4). Cross-sectional samples were prepared using a focused ion beam (FIB) instrument (Fig. 5b, c, e). Small parallelogram-shaped structures (side length 2.4 ± 0 .2 μm, 4.1 ± 0.3 μm) were observed after 30 min of reaction time (Fig. 3a). However, these structures were not present in samples analyzed after longer reaction times and that contained larger and elongated objects. These morphological changes were also accompanied by a structural rearrangement at the molecular level. Raman spectroscopy of the parallelogram-shaped structures (*t* = 30 min) and the final crystals (*t* = 48 h) showed clear differences (both shifts and signal shapes) in the ranges: *ν* = 950–1050 cm^−1^ (pyridine), *ν* = 1590–1620 cm^−1^ (pyridine), and *ν* = 1620–1650 cm^−1^ (C = C) (Supplementary Fig. 10). IR spectra show a signal intensity decrease at *ν* = 2750–3000 cm^−1^ (pyridine); during the course of the reaction a marked intense band appears at *ν* = 1660 cm^−1^ (pyridine) (Supplementary Fig. 11). These observations are consistent with Ostwald ripening^40^: small kinetically favored particles dissolve to form larger and thermodynamically favored structures. After 1.5 h, cylindrical-like crystals (vide supra) were formed having an apparent defect-free monodomain morphology and hexagonal-shaped termini (*l* = 29.9 ± 4.8 μm, *⌀* = 8.2 ± 2.0 μm) (Fig. 3b and Supplementary Fig. 9). The presence of a central concavity was visible at the termini. Micro-CT reconstruction revealed inner areas with relatively low electron densities and even empty regions (Fig. 4a and Supplementary Movie 2). After 3 h, the dimensions of the crystals increased both in length and diameter while maintaining a high level of uniformity (Fig. 3c and Supplementary Fig. 12). Enlarged cavities having diameters of ~1 μm were evident at the extremities. SEM analysis of the cross-sections, as well as the micro-CT data, showed that these cavities initially involved only the termini (Fig. 4b). Upon increasing the reaction times, the cavities propagated inside the cylindrical-like crystals, forming one double-cone-shaped channel (Fig. 4c, d). The diameter of these channels at the termini were ~15 μm after 48 h. Another striking morphological transformation was observed after 3 h: the channels were formed concurrently with the change in the external surface texture (Fig. 3b–e center images and Supplementary Figs. 2, 12, 13). The smooth surface gradually evolved into protruding areas by layered addition of materials (Supplementary Fig. 14), eventually resulting in six-connected half-rods after 48 h. These half-rods developed first in those areas that are closest to the termini via preferential deposition of material on the lateral edges (Supplementary Fig. 12, morphological feature highlighted by cyan and yellow lines).

Mechanistically some of the structural features (i.e., inner texture, a double-cone-shaped channel) suggest that material was redissolved during the growth process (Figs. 3 and 4). We assume that supramolecular objects undergo reorganization. The bond dissociation energy of nickel(II)–pyridine is ~43 kcal/mol and is prone to dissociation during heating^41^. The formation of the morphology seems strongly influenced by the balance between dissociation of weak bonds and redeposition. Plausible explanations are the presence of higher reactive regions (due to crystalline defects at the edges) and a higher degree of supersaturation (due to the diffusion of TPVA and metal salt from the dissolving crystal termini). The formation of the half-rods gradually progressed toward the center of the structure in parallel with the development of the double-coned-shaped channel. This multidomain shaping resulted in three structurally similar subdomains along each half-rod (clearly observed after 48 h). Linear engravings in the form of the main veins of leaves were evident after 24 h at the internal surfaces of the cavities close to the termini. Such features are typical of crystal erosion^42,43^. The crystal growth bears clear hallmarks of inside-out Ostwald ripening. The regular Ostwald ripening (~3 h) is followed by a relatively slow process whereby the initially formed defective monodomain crystals serve as both sacrificial templates and feedstock for the multidomain, single crystals.

### Sonication as a means to control crystal morphology and reactivity

We found that under solvothermal conditions, without sonication, monodomain hexagonal prism are formed that have a morphology and crystallographic structure that closely resembled the initial, sacrificial crystals (Supplementary Figs. 9 and 15). The diffraction (single-crystal X-ray, PXRD, TEM-single-crystal X-ray comparison, Supplementary Figs. 3 and 16 and Supplementary Note 2) and optical properties (Raman and FT-IR, Supplementary Figs. 10 and 11) were nearly identical^39^. Although these monodomain, single crystals are isomorphous to the crystals obtained using sonochemical–solvothermal conditions (*t* = 1.5 h), they did not develop further into the multidomain structures. Micro-CT measurements of the hexagonal prisms (*l* = 9.9 ± 0.5 μm, *⌀* = 4.3 ± 0.4 μm) having six slightly bent facets (*t* = 48 h) indicated the formation of defect-free, solid crystals (Supplementary Fig. 17). The density distribution is nearly constant and no empty regions are observable. Ex situ SEM measurements of these solid crystals were carried out by stopping the solvothermal reaction at different reaction times (Supplementary Fig. 18). The measurements indicated an Ostwald ripening from parallelogram to prismatic structures and highly uniform crystals—akin to the formation of the initial crystals formed under sonication–solvothermal conditions. The main mechanistic difference between the solvothermal and sonication–solvothermal conditions that we observed was the reactivity of the parallelogram-shaped structures formed at the beginning of the reactions (*t* = 30 min).

Sonication–solvothermal conditions accelerate the dissolution of parallelogram-shaped crystals to form defective cylindrical crystals as observed by micro-CT (Fig. 4a and Supplementary Movie 2) that further transferred into the hollow and multidomain single crystals. At a much earlier stage, the formation of elongated crystals was observed by using sonicated solvent (sonication–solvothermal: ~1.5 h versus solvothermal: ~24 h) (Fig. 3 and Supplementary Figs. 18–20). Sonication of the solvent prior to the solvothermal reaction is the key to the formation of the multidomain, single crystals. To further verify this observation, isolated parallelogram crystals obtained under solvothermal conditions were added to the sonicated solvent and converted to the hollow and multidomain single crystals (albeit also other structures were observed; Supplementary Fig. 21). Likewise, isolated parallelogram crystals obtained under sonochemical–solvothermal conditions were added to non-sonicated solvent and formation of the monodomain, hexagonal prism were observed (Supplementary Fig. 22).

EPR measurements were carried out after the sonication of the solvent mixture; they showed the formation of DMF radicals having a lifetime of ~45 min at room temperature, even in the presence of NiBr_2_ (Supplementary Fig. 1)^33,34^. Such metal salts are known to interact with analogous radical species^44^. In order to further elucidate the role of the sonication pretreatment, we hypothesized that radicals (or products therefore) could reduce the amount of reactive NiBr_2_. Indeed, the formation of hollow, multidomain crystals have been observed by SEM when lowering the concentration of NiBr_2_ by 50% or more under solvothermal conditions after 48 h (Supplementary Fig. 23). An excess of metal salt is known to stabilize and direct the formation of organic crystals with ligands that are structurally similar to TPVA^29^. Inactivating pyridine coordinating sites by excess of metal salts can be the cause of the here observed differences in growth kinetics (Fig. 3 and Supplementary Figs. 18–20). The faster growth kinetics of the unstable, cylindrical structures (sonochemical–solvothermal conditions, *t* = 1.5 h) is consistent with the presence of low density inner regions. Saturation of the crystal surface by the metal salt can have a stabilizing effect, hence the Ostwald ripening (excess of metal) versus the inside-out Ostwald ripening pathways.

Thermogravimetric analysis (TGA) under nitrogen reveals similar thermal stabilities of the prismatic crystals separated under the solvothermal conditions and the hollow and multidomain single crystals synthesized under sonication–solvothermal conditions (for both crystals, *t* = 48 h). These observations indicate that the stability of the isostructural crystals is related to the 3D metal-organic networks, and not affected by the different morphology or defects (Supplementary Fig. 24).

## Discussion

Single-crystallinity emerges first, whereas the multidomain and curved appearance occurs afterwards. The complexity of the formation includes different processes that are dominant during specific time periods. Prismatic crystals formed at relatively early stages undergo a series of transformations (dictated by factors such as metal salt concentration, structural defects, and local supersaturation changes), finally resulting in the multidomain, single crystals. Despite the complex molecular packing, the crystallographic structure neither changes nor develops polycrystallinity properties during the single-to-multidomain morphological transformation. Our new findings confirm the idea that such chiral, metallo-organic crystals are not oddities, but rather are among the first examples of a new class of paradoxical and isomorphous materials with concurrently extraordinary shapes and sought-after crystalline properties (e.g., single-crystallinity, porosity, and chirality)^30^. Our previously reported *yoyo*-shaped, single crystals are formed from a copper nitrate salt and a poorly soluble ligand^30^, structurally similar to the here reported tetrahedral achiral ligand (having carbon–carbon triple bonds instead of double bounds). Despite differences in bond order and solubility, these crystals are isostructural and belong to the rare *P*622 space group. These findings might indicate that the octahedral molecular geometry around the metals center having four pyridine groups in plane is a key factor to the molecular packing of such crystals, whereas their complex and highly unusual morphologies are determined mainly by other factors including metal-to-ligand ratios, reaction time, and growth kinetics. Sonochemistry has been used for the formation of MOFs^45,46^. Typically, the organic constituent and metal salt are mixed together in a solvent and exposed to high-energy ultrasounds. The product formation has been related to local increases of temperature and pressure by implosion of cavitation vacuum bubbles. Our finding indicates that sonication can also change the concentration and ratios of the active components resulting in morphologically different structures.

## Methods

### Materials

Chloroform (CHCl_3_, ≥99.8%) and DMF (≥99.8%) were obtained from Sigma Aldrich and J.T. Baker, respectively. NiBr_2_ (>98.0%) was obtained from Sigma Aldrich. Reagents were used without further purification. 5,5-Dimethyl-1-pyrroline *N*-oxide (DMPO) (>98.0%) was obtained from Cayman Chemical Co. and used as a spin trap. Glass pressure tubes (Ace Glass, Inc., pressure tubes #15 with a plunger valve, PTFE Bushing and FETFE^®^ O-Ring, volume 50 ml) were cleaned by immersion in a H_2_SO_4_/30% H_2_O_2_ piranha solution (7:3 v/v) for 10 min. Subsequently, they were washed with deionized water and dried in an oven for 24 h at 130 °C. Caution: piranha is an extremely dangerous oxidizing agent and should be handled with care using appropriate personal protection. 1,3,5,7-Tetrakis{4-[(E)-2-pyridin-4-yl-vinyl]phenyl} adamantane (TPVA) was prepared according to a literature procedure^47^.

### Preparation of the MOFs

Sonochemical–solvothermal crystallization: a mixture of CHCl_3_ (1.0 ml) and DMF (2.0 ml) was sonicated in a glass vial (20 ml) with a DCG-120H MRC professional ultrasonic cleaner in normal mode (33–40 KHz frequency) for 1.5 h in an ice-bath. Subsequently, this solution was used immediately to dissolve TPVA (3.0 mg, 3.5 mmol, conc. 1.2 mM). The metal salt was dissolved in DMF (5.0 mg of NiBr_2_ in 3.3 ml DMF, metal salt conc. = 7.0 mM). The solution of TPVA (3.0 ml, 3.5 mmol) and 1.0 ml of the solution of the metal salt (7.0 mmol) were mixed in a glass pressure tube (the final concentrations of TPVA and the metal salt in the solution are 0.9 and 1.8 mM, respectively). Then, the tube was sealed and heated in an oven at 105 °C for 48 h without stirring and with the exclusion of light. Next, the tube was removed from the oven and left at room temperature for 5 min before opening. A green precipitate was formed; it was isolated by centrifugation and washing with ethanol.

Solvothermal crystallization: this procedure is identical to the above-described sonochemical–solvothermal crystallization process but without sonication of the solvents for 1.5 h in an ice-bath.

### Scanning electron microscopy (SEM) and focused ion beam (FIB)-assisted cutting

SEM measurements were performed using HRSEM ULTRA-55 ZEISS and SIGMA 500 ZEISS instruments at an EHT voltage of 1.5 kV. Images were collected in secondary mode by using an Everhart–Thornley detector. Samples were prepared by placing a drop of the reaction mixture on a silicon substrate and allowing the solvent to evaporate. A Helios 600 FIB/SEM dual beam microscope was used for the assisted cutting and the subsequent imaging of the structures. In order to immobilize the structures during the cut, beam-assisted deposition of platinum layers was performed between the sides of the structures and the silicon substrate before milling.

### Transmission electron microscopy (TEM)

TEM samples were prepared by placing 5 µl drops of the reaction mixture on 400-mesh carbon-coated copper grids (SPI-Grids^TM^, 3 mm) followed by blotting after 10 s. TEM nanobeam scanning electron diffraction was recorded under low-dose conditions with a FEI Tecnai F20 Twin TEM in STEM microprobe mode with a defocused beam of ~10 nm diameter and at a beam current of 10 pA. Undersampling was applied in order to avoid beam damage causing lattice damage during the exposure of 0.1 s per raster point. For a comparison with the crystal structure obtained by single-crystal X-ray diffraction, electron diffraction patterns were simulated using SingleCrystal (CrystalMaker Software Ltd., UK).

### Micro-computed tomography (Micro-CT)

The MOFs were separated from the mother liquor by centrifugation and dried overnight under vacuum by a vacuum pump (Edwards RV12). A plastic pipette tip was used as a sample container: the narrowest extremity of the pipette tip was melted and sealed using a flame. Subsequently, the dried crystals were placed into the tip. Micro-CT data of the MOF obtained by the sonochemical–solvothermal crystallization were acquired with a Micro-XCT400 Zeiss X-ray microscope (Peasanton, California, USA). The tomographic images were obtained by taking 1200 projections over 180 deg at 40 KV and 200 µA. The final pixel sizes were 0.33 µm. Micro-CT data of the MOFs obtained by the solvothermal crystallization were acquired with a Xradia 520 Versa Zeiss X-ray microscope. The tomographic images were obtained by taking 2401 projections over 360 deg at 100 KV and 90 µA. The final pixel size was 0.39 µm. 3D images of the samples were collected for all the analyzed systems. Finally, several individual structures were analyzed in detail by using the Avizo 9.5 software (Thermo Fisher Scientific Inc, USA).

### Single-crystal X-ray diffraction (SXRD)

SXRDs were collected both by a synchrotron source at the Beamline ID-29 of the European Synchrotron Radiation Facility and by a Rigaku XtaLab^Pro^ X-ray home-source diffractometer. The XtaLab^Pro^ X-ray diffractometer is equipped with a four-circle Kappa goniometer, a Dectris S200K detector, and a micro-focus sealed tube with microCMF-VHF. The data were collected with *λ* = 0.700 Å (synchrotron) and for CuKα1 radiation, *λ* = 1.5418 Å (Rigaku XtaLab^Pro^ diffractometer). The crystals were placed in Hampton Paratone oil, mounted on a MiTeGen loop, and plunged into liquid nitrogen to flash freeze them. The data were collected at 100 K with Oxford Cryostream. The crystals analyzed at the synchrotron were transported frozen in a Taylor-Wharton CX100 dry shipper. Data collection and reduction for the synchrotron data were done using MXCube, and using the EDNA automated data processing pipeline with XDS. Data collection, reduction, and analysis for the XtaLabPro laboratory data were performed with the CrysAlisPro software package (version 1.171.39.22a). The crystal structures were solved by direct methods using SHELXT 2016/4^48^. All non-hydrogen atoms were further refined by SHELXL with anisotropic displacement coefficients. Hydrogen atoms were assigned isotropic displacement coefficients, and their coordinates were allowed to ride on the respective carbon atoms. The Platon SQUEEZE protocol was applied for all the structures^49,50^. Mercury CSD 3.10.2 and PLATON software were used for graphics. The reported Ewald sphere’s projections were generated by the Rigaku XtaLabPro instrument with CrysAlisPro, from diffraction data taken with a 100 μm beam; such a beam is large enough to expose the entire crystal. Details of the crystal structure analysis are presented in Supplementary Table 1.

### Powder X-ray diffraction (PXRD)

Diffraction measurements were carried out by reflection geometry using an Ultima III (Rigaku, Japan) diffractometer equipped with a sealed Cu anode X-ray tube operating at 40 kV and 40 mA. A bent graphite monochromator and a scintillation detector were aligned in the diffracted beam. Next, *θ*/2*θ* scans were performed under specular conditions in the Bragg–Brentano mode with variable slits. The samples were scanned from 5 to 30 degrees in step mode with a step size of 0.025 degrees and a collection time of 12 s per step. The phases the XRD pattern were analyzed using Jade 2010 software (Materials Data, Inc.). The lattice parameters were refined using the Whole Pattern Fitting/Rietveld refinement module of Jade 2010.

### Micro-raman spectroscopy

Raman measurements were performed on a LabRAM HR Evolution instrument (Horiba, France), equipped with an 800 mm spectrograph and a CCD detector (1024 pixels × 256 pixels open electrode front illuminated CCD camera, cooled to −60 °C). The system was based on an open confocal microscope (BX-FM Olympus, Japan). A 632.8 nm HeNe laser, with 600 grooves/mm grating and a ×100 objective (with spatial resolution better than 1 μm) were used for the measurements. The pixel spacing was 1.3 cm^−^^1^.

### Electron paramagnetic resonance (EPR)

EPR spectra were recorded on a Bruker ELEXSYS 500 X-band spectrometer equipped with a Bruker ER4102ST resonator in a Wilmad flat cell for aqueous solutions (WG-808-Q) at room temperature. The experimental conditions were 1024 points, with a microwave power of 20 mW, 0.1 mT modulation amplitude, and 100 kHz modulation frequency. The sweep range was 10 mT. The spectrum was simulated using a custom-written MATLAB program based on EasySpin subroutines.

### Elemental analyses

Elemental analyses (C, H, N, Cl, Ni, Cl, Br) were performed at Kolbe Laboratorium, Mulheim, Germany. The oxygen is calculated as the leak to 100%. The errors are ±0.01% for C, H, N; ±0.015% for Ni, and ±0.005% for Cl and Br.

### Thermogravimetric analysis (TGA)

TGA was performed by a SDT Q600 V8.3 Build 101 Instrument. MOF-NiBr_2_ obtained under sonochemical–solvothermal and solvothermal conditions (*t* = 48 h, 105 °C) were dried under vacuum overnight and put in an alumina sample pans under a stream of nitrogen for the analysis (30–800 °C).

### Circular dichroism (CD)

CD and relative UV spectra were collected by a Chirascan™—plus Auto CD Spectrometer. The measurements were performed over a wavelength range of 200−600 nm, by using a 1 mm path length cuvette. The spectral resolution was 1 nm.

## Supplementary information

Supplementary Information

Description of Additional Supplementary Files

Supplementary Movie 1

Supplementary Movie 2

## Data Availability

The data supporting the findings of this study are available within the paper and its Supplementary information files. The crystallographic data have also been submitted to the database at the Cambridge Crystallographic Data Centre. The CCDC numbers are 1996729 and 2016190. CIF V296 corresponds to Fig. 5d and Supplementary Figs. 5 and 8, left panel. CIF V394 corresponds to Supplementary Figs. 6 and 8, right panel.
