# Supplementary material for: Molecular cannibalism: Sacrificial materials as precursors for hollow and multidomain single crystals
Source: Nat Commun. 2021 Feb 11;12:957. doi: 10.1038/s41467-021-21076-9 (PMC7878748; doi:10.1038/s41467-021-21076-9)
Supplement: Supplementary file 1 — Supplementary Information [file 41467_2021_21076_MOESM1_ESM.pdf]

# Supplementary Information

# Molecular Cannibalism: Sacrificial Materials as Precursors for Hollow and Multidomain Single Crystals

di Gregorio et al.

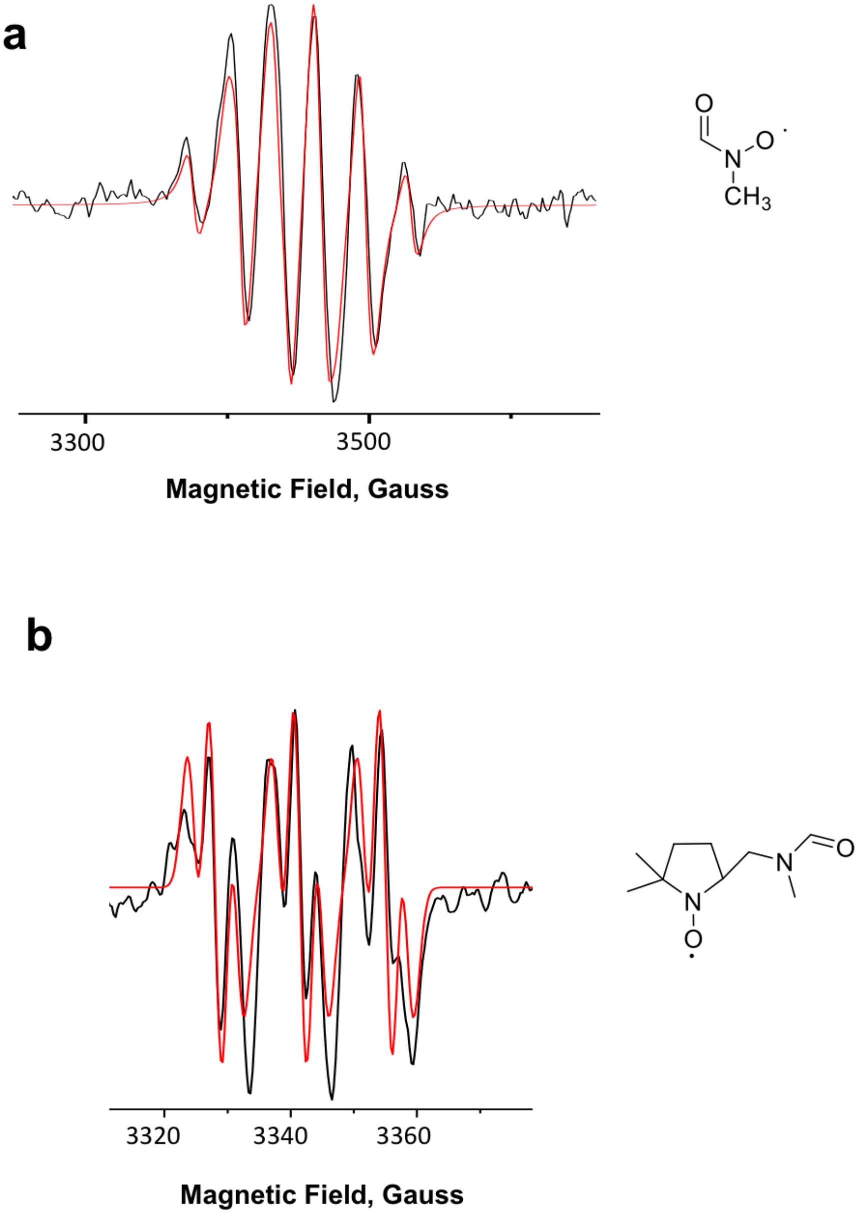


**Supplementary Fig. 1.** Experimental (black line) and simulated (red line) electron paramagnetic resonance (EPR) spectra of $CHCl_3$ (1.0 mL)/DMF (2.0 mL) that was sonicated for 1.5 h **a** without an additive and **b** in presence of a spin trap (5,5-dimethyl-1-pyrroline *N*-oxide, DMPO). The radical species resulting from the spectra analysis are shown on the right.[1-3]

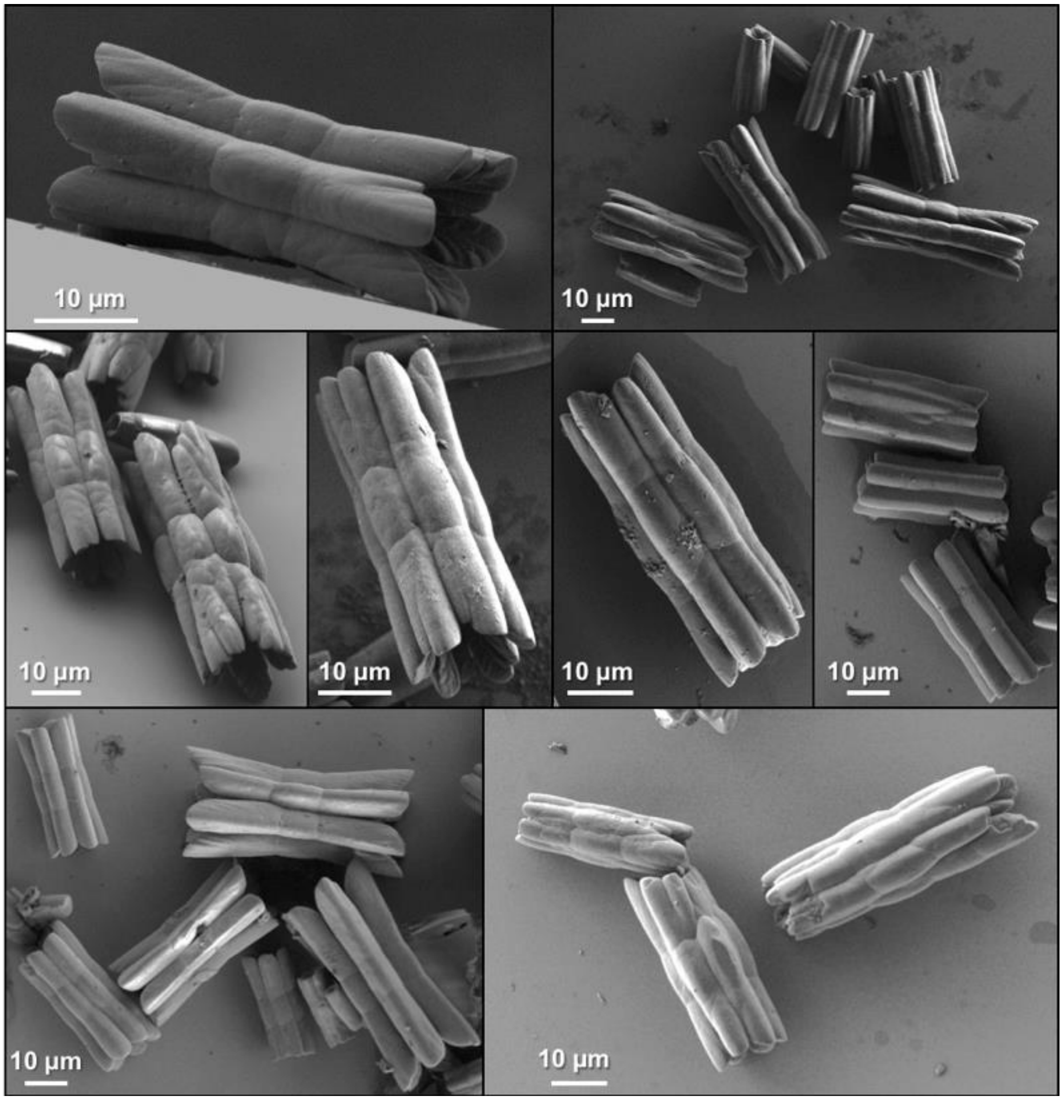


**Supplementary Fig. 2.** Scanning electron microscopy (SEM) images of **MOF-NiBr$_2$** (sonochemical-solvothermal conditions, $t$ = 48 h).

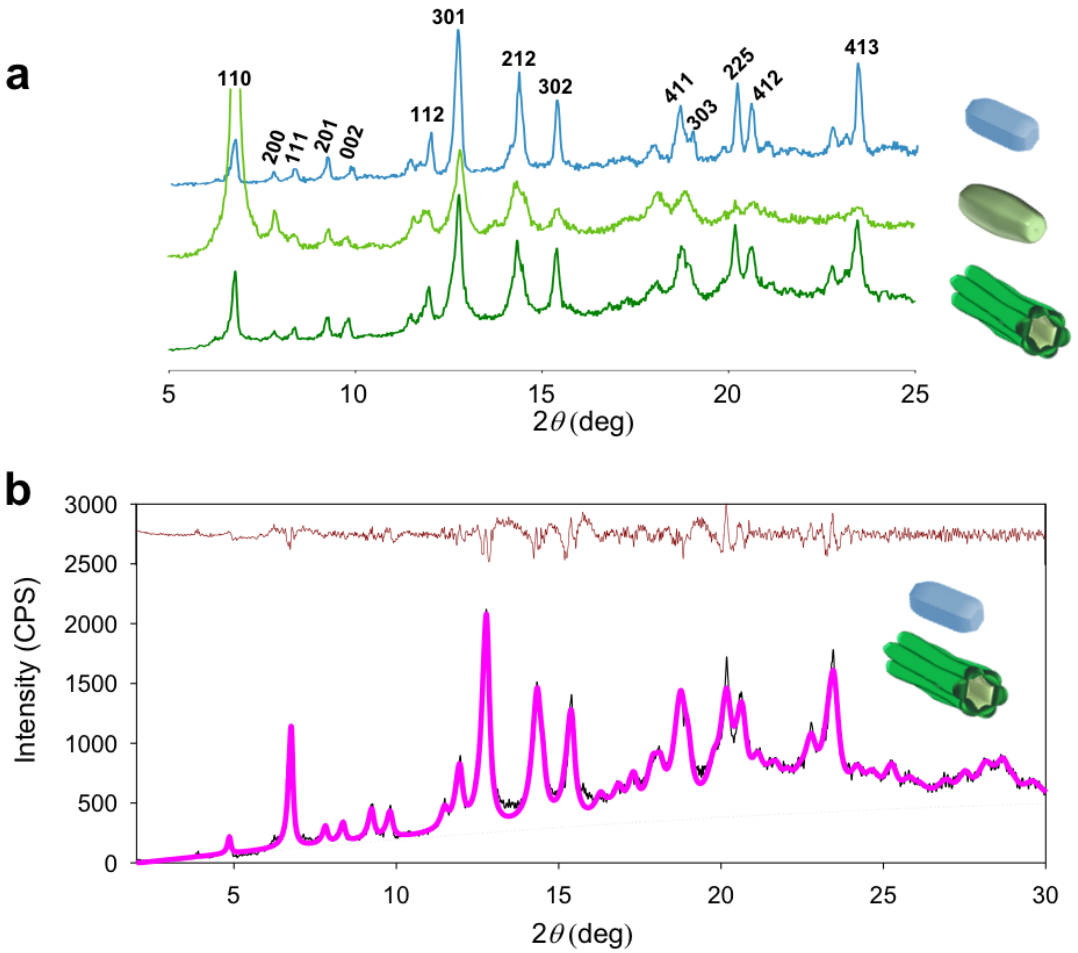


**Supplementary Fig. 3. a** Experimental powder X-ray diffraction (PXRD) spectra of **MOF-NiBr**$_2$: solvothermal conditions, 48 h (top), sonochemical-solvothermal conditions, 1.5 h (center), and 48 h (bottom). **b** The experimental PXRD spectrum of **MOF-NiBr**$_2$, sonochemical-solvothermal conditions, $t$ = 48 h (black line) was fitted (purple line) by using the single-crystal X-ray data of **MOF-NiBr**$_2$, solvothermal conditions, $t$ = 48 h, (CCDC 1965786).[4] The difference between the fitted and experimental curves is reported at the top of the spectra (brown lines). The base line is denoted as a dotted line. The goodness-of-fit is 1.96. Best fitting values of the unit cell dimensions are a = b = 25.86(1) and c = 17.90(9). Experimental intensity variation due to preferred orientation was considered in the fit by using spherical harmonic functions[5] (Jade 2010).

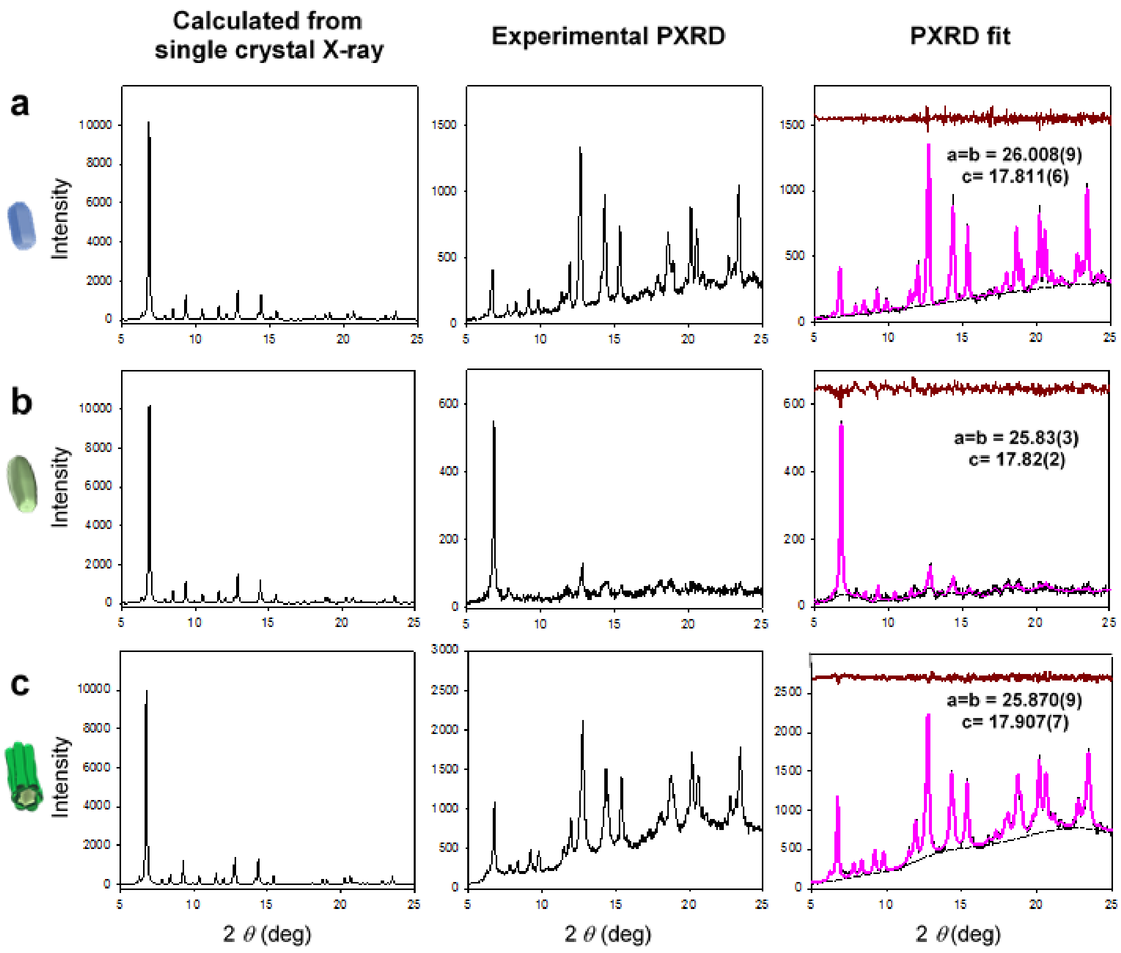


**Supplementary Fig. 4.** Left: Powder X-ray diffraction (PXRD) spectra calculated from the single-crystal X-ray data (CCDCs 1965786,[4] 1996729 and 2016190). Center: experimental PXRD curves for **MOF-NiBr$_2$**: (**a**) solvothermal conditions, $t$ = 48 h, (**b**) sonochemical-solvothermal conditions, $t$ = 1.5 h, and (**c**) and sonochemical-solvothermal conditions, $t$ = 48 h (**c**). (Right) The experimental PXRD curves were fitted (purple lines) by using the corresponding single crystal X-ray data. The difference between the fitted and experimental curves is reported at the top of the spectra (brown lines). The base line is denoted as dotted line. The goodness-of-fit are 1.26, 1.33 and 1.67. Best fitting values of the unit cell dimensions are shown in the spectra. Experimental intensity variation due to preferred orientation was considered in the fits by using spherical harmonic functions[5] (Jade 2010).

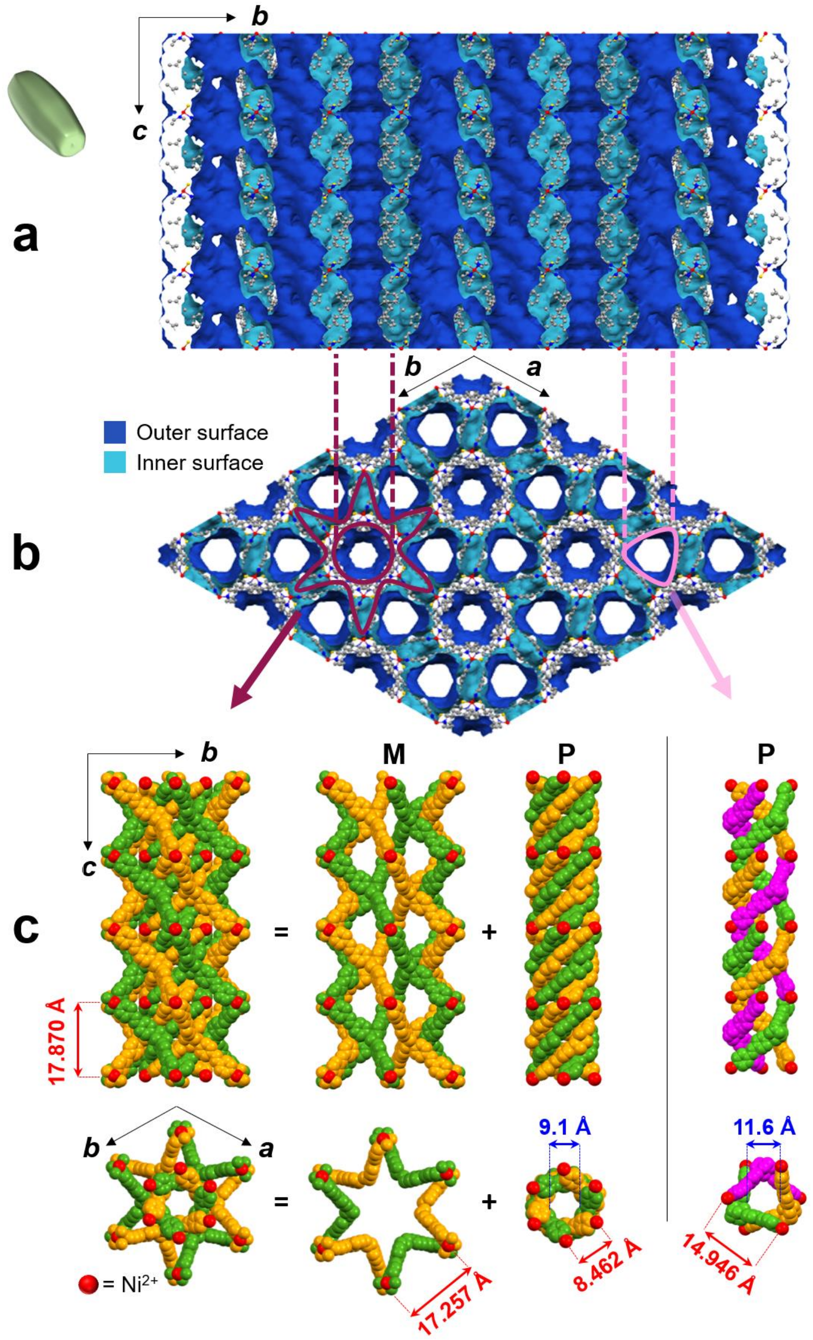


**Supplementary Fig. 5.** Single-crystal X-ray data of **MOF-NiBr$_2$** (sonochemical-solvothermal conditions, 1.5 h) (CCDC 1996729). **a** Connolly surface representation of the crystal structure down the a and c axes. **b**. Top view of the channels having hexagonal ($\varnothing \approx 9.1$ Å) and triangular ($\varnothing \approx 11.6$ Å) geometries, denoted by bordeaux and pink lines, respectively. The diameters are reported without taking the hydrogens into account. **c** Helicoidal structure of the channels. The helicates constituting the channels are denoted in orange-green and orange-green-magenta for the hexagonal and triangular channels, respectively.

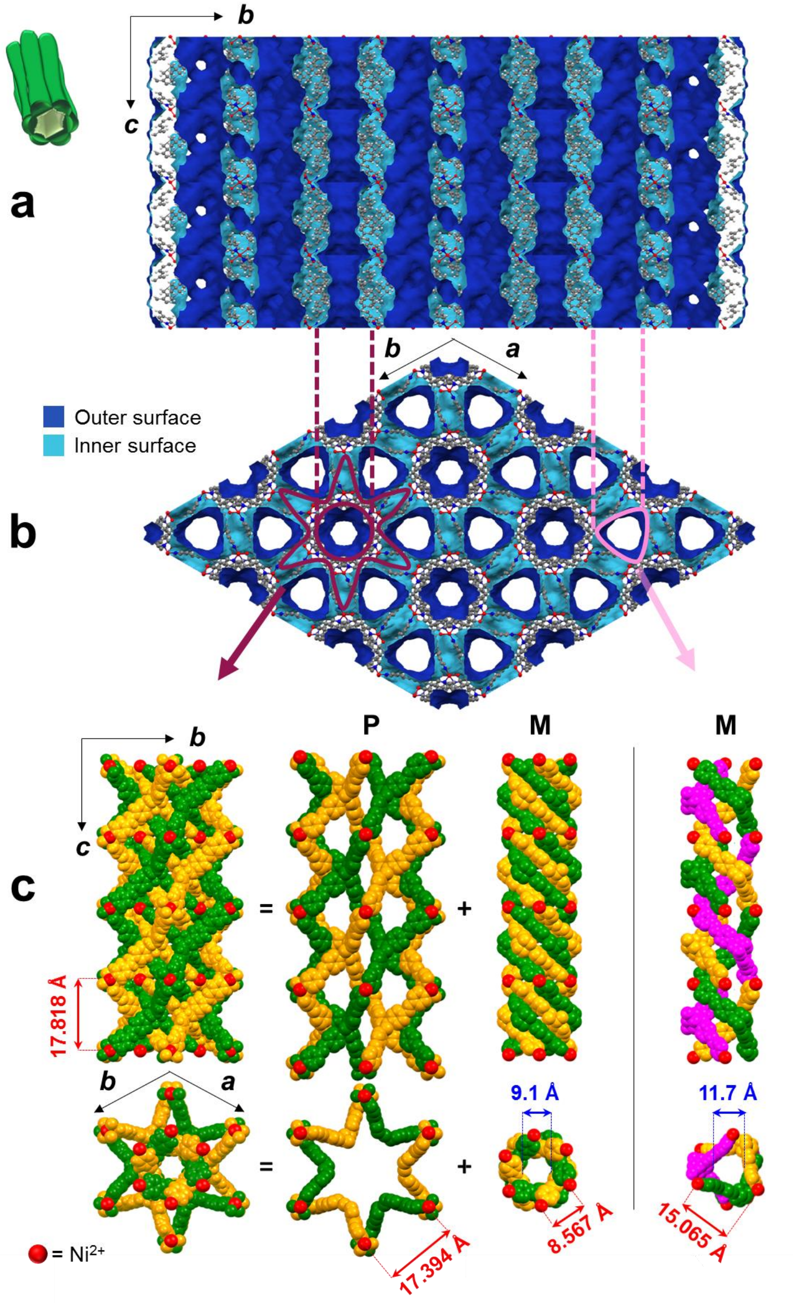


**Supplementary Fig. 6.** Single-crystal X-ray data of hollow, multidomain **MOF-NiBr$_2$** (sonochemical-solvothermal conditions, 48 h) (CCDC 2016190). **a** Connolly surface representation of the crystal structure down the a and c axes. **b**. Top view of the channels having hexagonal (⌀ ≈ 9.1 Å) and triangular (⌀ ≈ 11.7 Å) geometries, denoted by bordeaux and pink lines, respectively. The diameters are reported without taking the hydrogens into account. **c** Helicoidal structure of the channels. The helicates constituting the channels are denoted in orange-green and orange-green-magenta for the hexagonal and triangular channels, respectively.

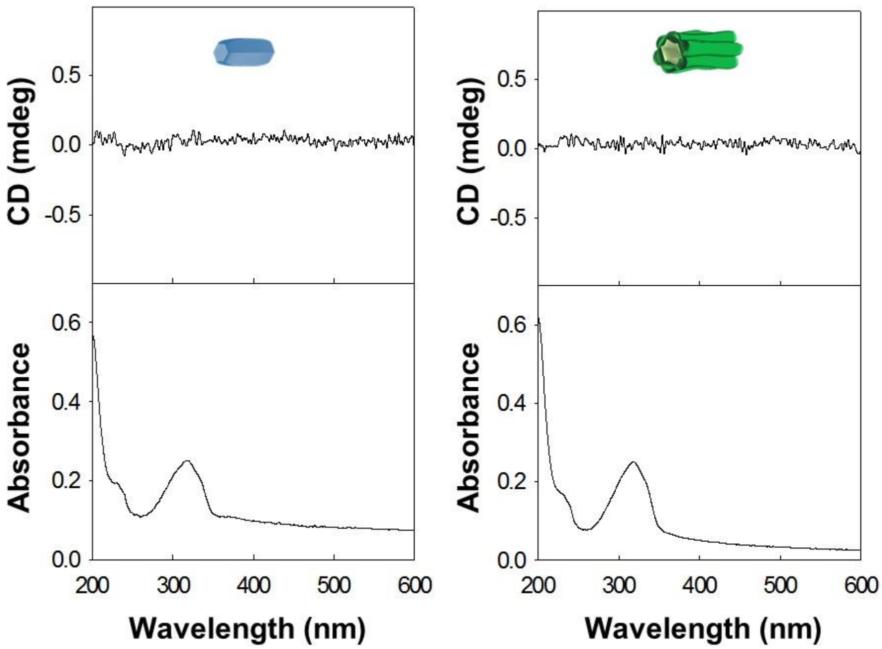


**Supplementary Figure 7.** Circular dichroism (top) and UV/Vis (bottom) spectra of **MOF-NiBr$_2$** obtained under solvothermal conditions (left) and sonochemical-solvothermal conditions (right) ($t$ = 48 h, 105 °C). The crystals were grinded and dispersed in ethanol.

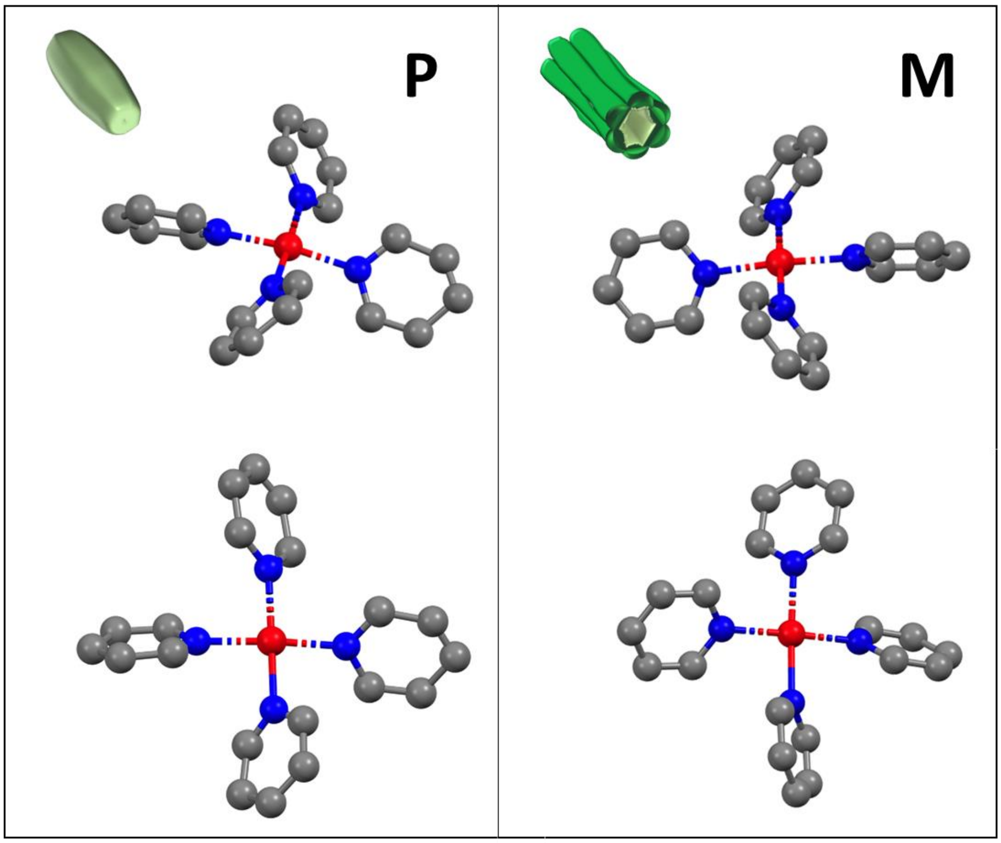


**Supplementary Fig. 8.** The propeller-type arrangement of the pyridine moieties around the Ni centers in the coordination nodes for **MOF-NiBr$_2$** obtained by sonochemical-solvothermal conditions, (left) $t$ = 1.5 h (CCDC 1996729) and (right) $t$ = 48 h (CCDC 2016190). Two perspectives of each of the coordination centers are shown. Oxygen atoms in axial positions were omitted for the sake of clarity. Color code: C gray, Ni red, N blue. Pyridine rings located on opposite sites within the coordination center have slightly different Ni–N distances: 2.021(15) Å and 2.037(14) Å for the crystal after $t$ = 1.5 h (left); 2.100(8) Å and 2.08(11) for the crystal after $t$ = 48 h (right).

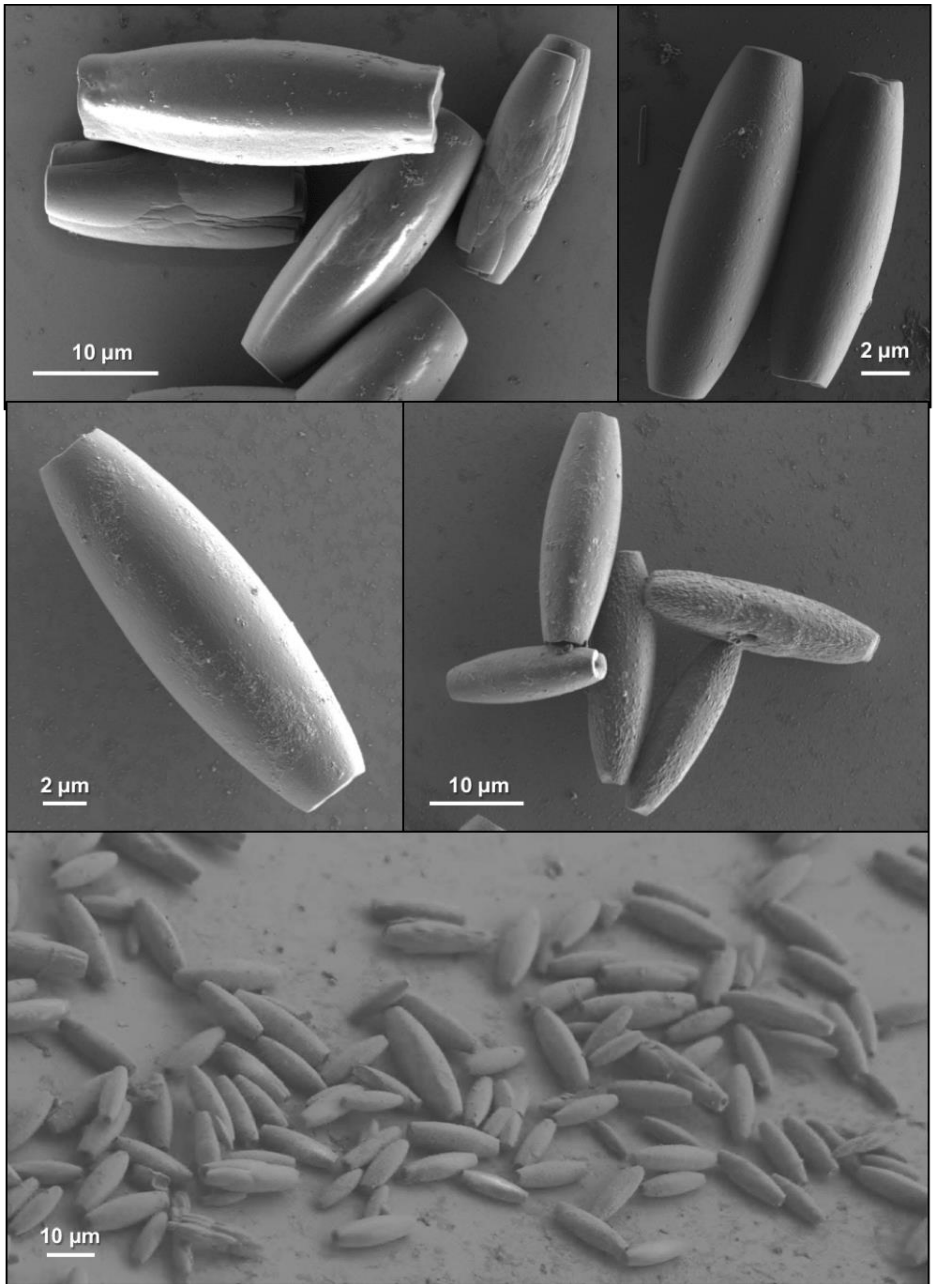


**Supplementary Fig. 9.** *Ex-situ* scanning electron microscopy (SEM) images of **MOF-NiBr$_2$** obtained under sonochemical-solvothermal conditions ($t$ = 1.5 h).

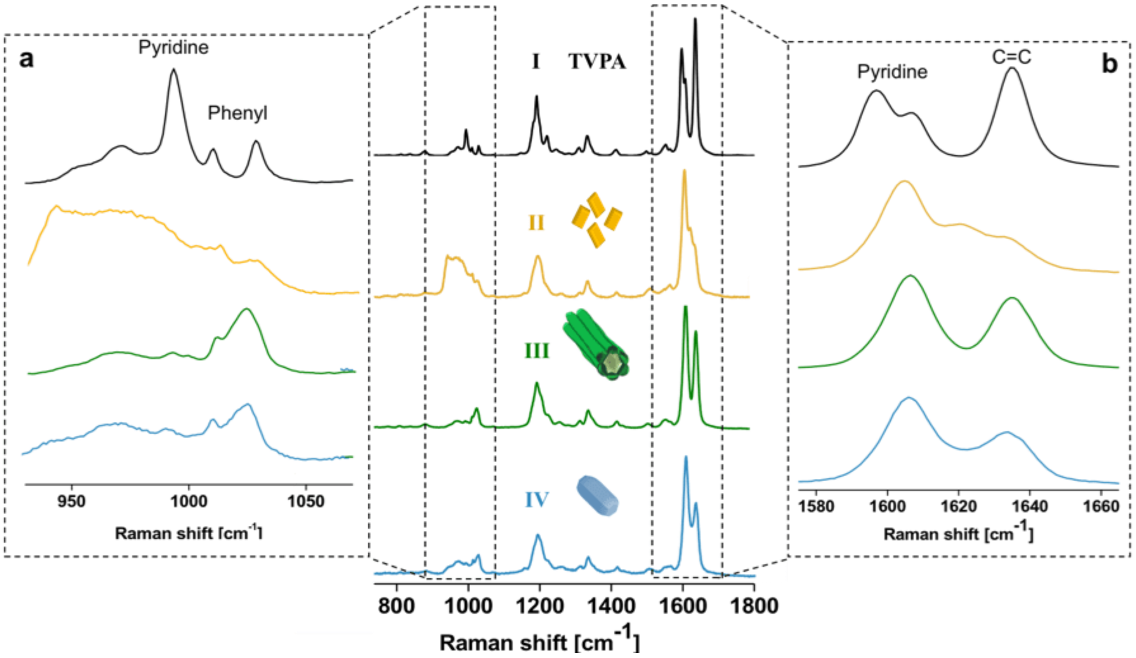


**Supplementary Fig. 10.** Raman spectra of (I) **TVPA**, and of the structures obtained by reacting **TVPA** with $NiBr_2$: (II) parallelogram-shaped structures, sonochemical-solvothermal conditions, *t* = 30 min. (III) hollow **MOF-NiBr$_2$**, sonochemical-solvothermal conditions, *t* = 48 h. (IV) **MOF-NiBr$_2$**, solvothermal conditions, *t* = 48 h.[4] **a,b** Raman spectra showing the regions: **a**. ν = 900-1100 $cm^{-1}$ and **b**. ν =1570-1670 $cm^{-1}$.

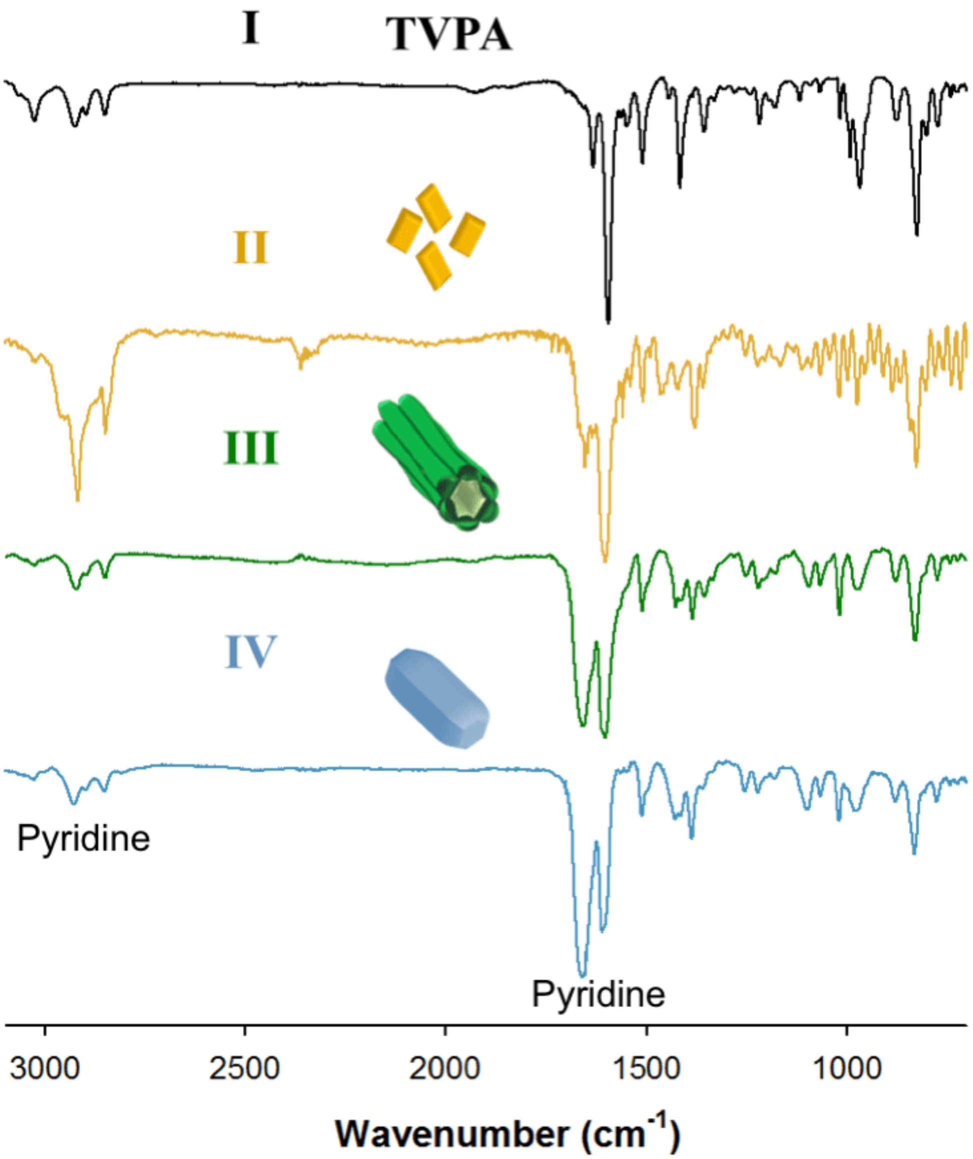


**Supplementary Fig. 11.** FT-IR spectra of (I) **TVPA**, and of the structures obtained by reacting **TVPA** with $NiBr_2$: (II) parallelogram-shaped structures, sonochemical-solvothermal conditions, $t$ = 30 min. (III) hollow **MOF-NiBr$_2$**, sonochemical-solvothermal conditions $t$ = 48 h. (IV) **MOF-NiBr$_2$**, solvothermal reaction conditions, $t$ = 48 h.[4] The samples were dried overnight under vacuum before the measurements.

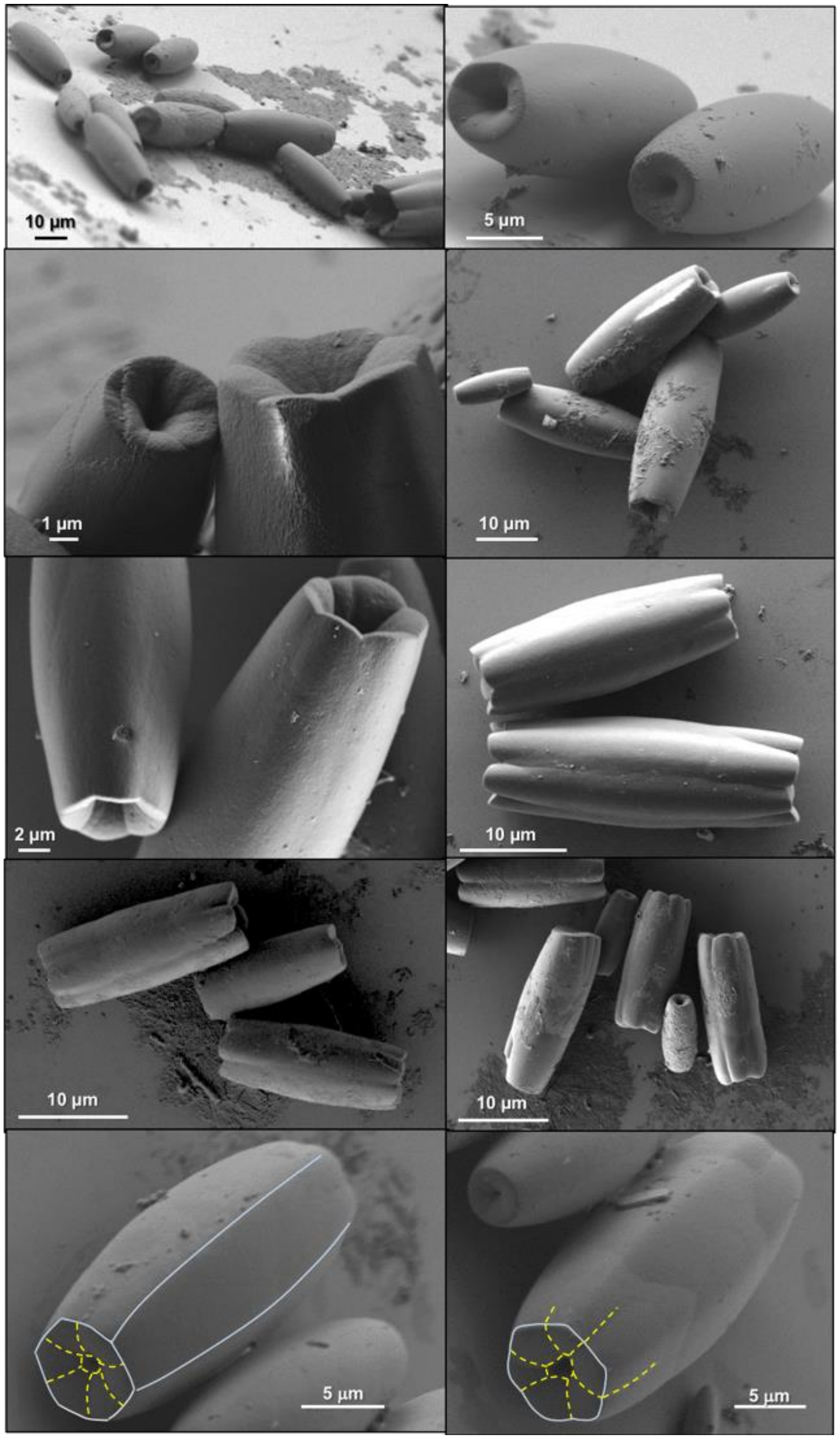


**Supplementary Fig. 12.** *Ex-situ* scanning electron microscopy (SEM) images of **MOF-NiBr$_2$**, sonochemical-solvothermal conditions, $t$ = 3 h. Cyan and yellow lines highlight properties of the initial prismatic morphology and overgrowing multidomain texture, respectively.

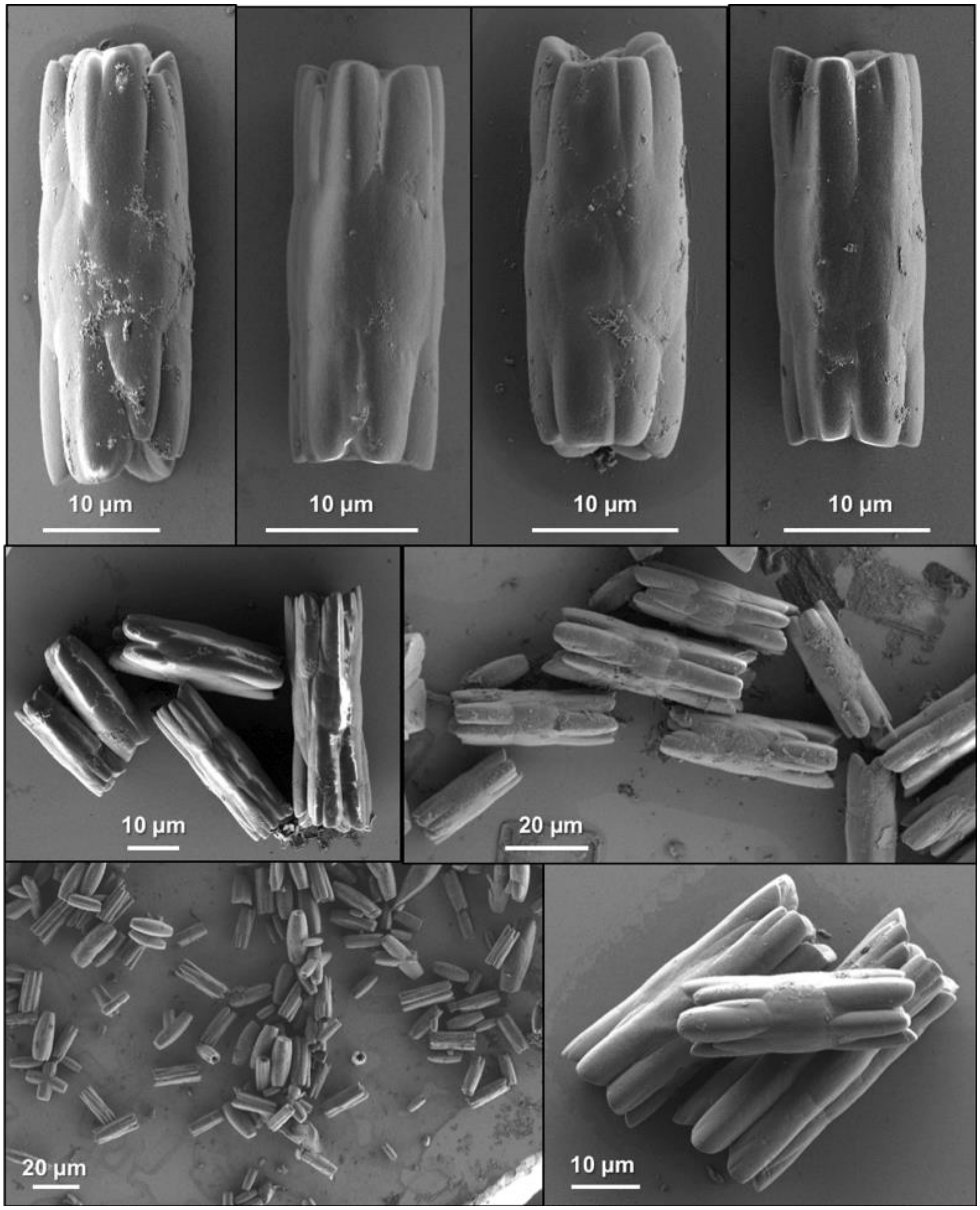


**Supplementary Fig. 13.** *Ex-situ* scanning electron microscopy (SEM) images of **MOF-NiBr$_2$**, sonochemical-solvothermal conditions, $t$ = 24 h.

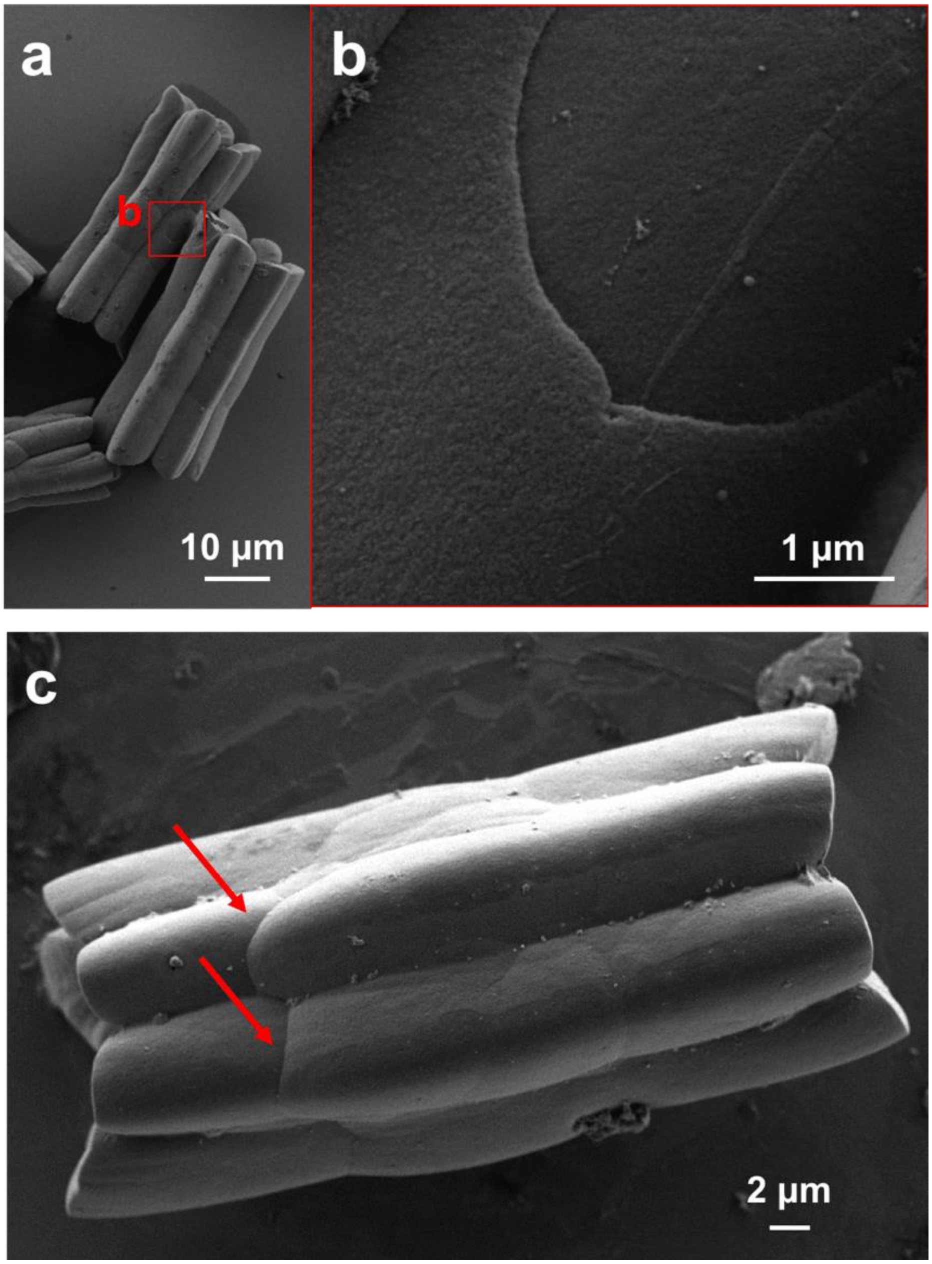


**Supplementary Fig. 14. a-c** Scanning electron microscopy (SEM) images of **MOF-NiBr$_2$**, sonochemical-solvothermal conditions, *t* = 48 h. **b** is the magnification of the area highlighted in **a** as red square. Arrows in **c** points to the layers.

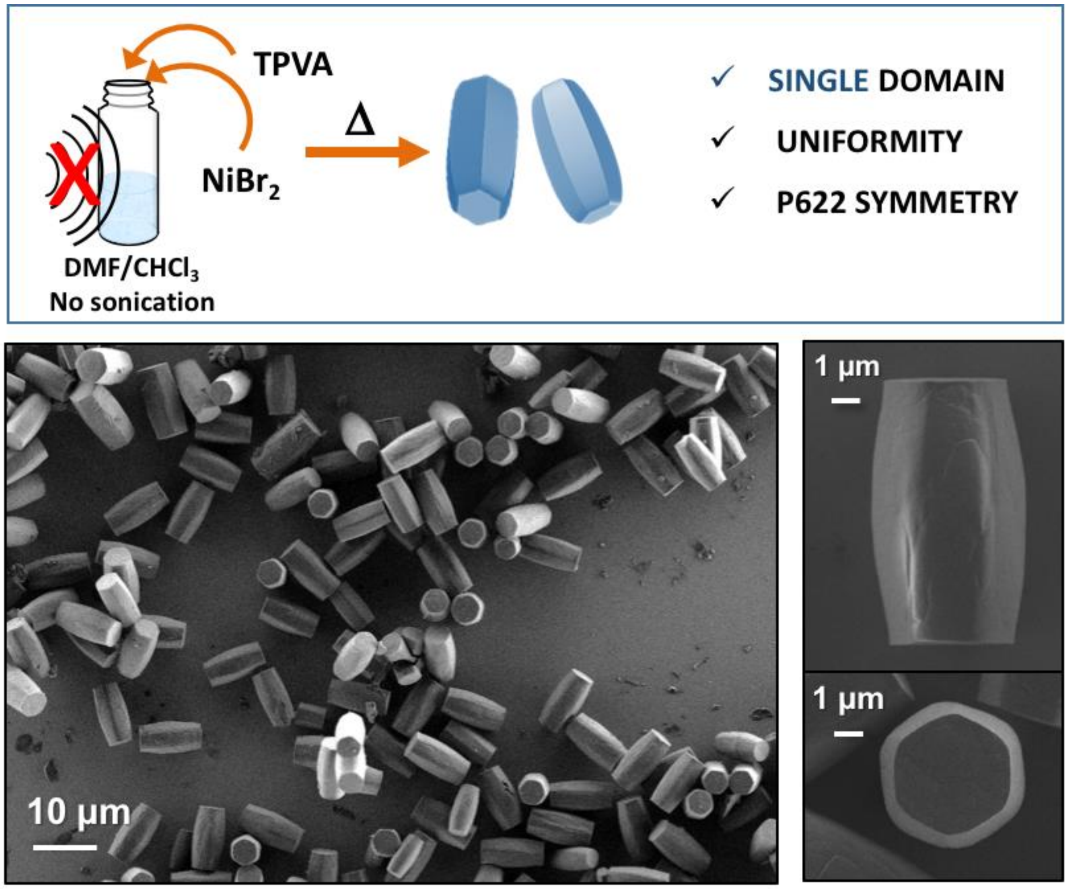


**Supplementary Fig. 15.** Crystals of **MOF-NiBr$_2$** obtained by reacting the organic ligand (**TPVA**) with $NiBr_2$, solvothermal conditions (105 °C, $t$ = 48 h) in DMF:$CHCl_3$ = 3:1 (v/v) (top).[4] Scanning electron microscopy (SEM) images of the prismatic structures (bottom).

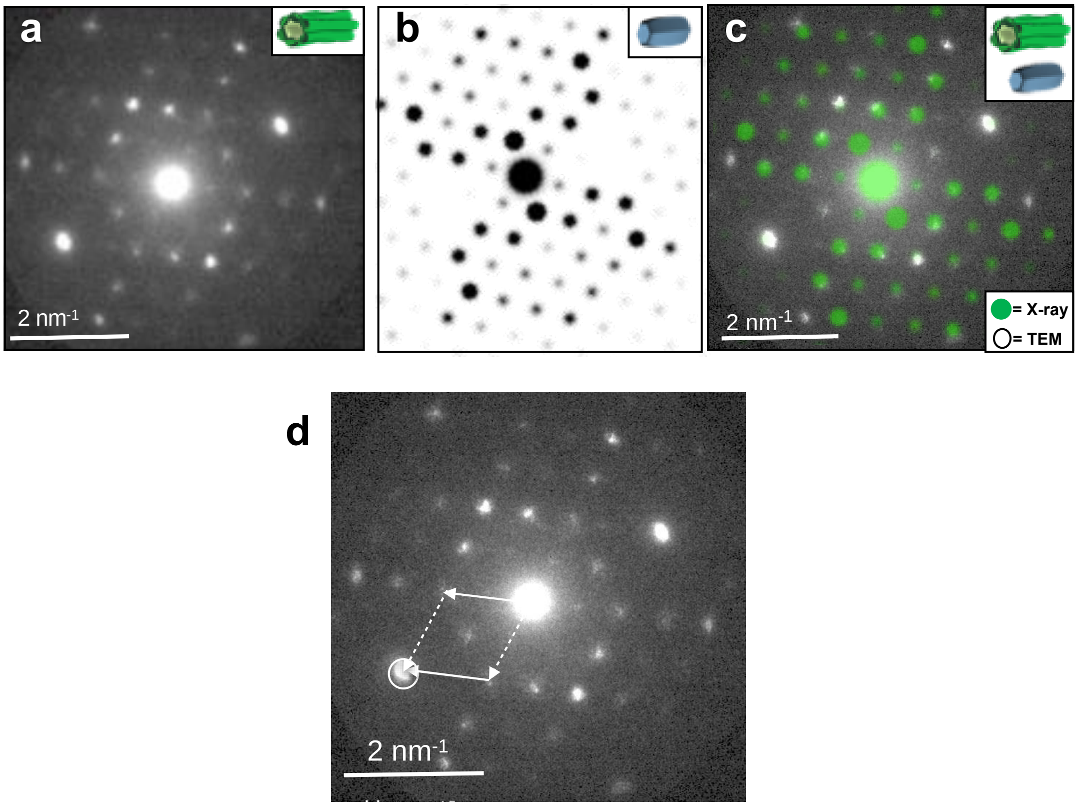


**Supplementary Fig. 16. a** Nano-beam electron diffraction of **MOF-NiBr$_2$** (sonochemical-solvothermal conditions, $t = 48$ h). **b** Kinematical $\langle 1\bar{1}\bar{1} \rangle$ zone axis patterns calculated from the single-crystal X-ray data of the prismatic **MOF-NiBr$_2$** (solvothermal conditions, $t = 48$ h),[4] using electron scattering factors. **c** Comparison between the diffraction data shown in **a** and **b**. Deviating intensities are related to dynamical scattering into reflections that are a sum of reflections in the same zone axis pattern. An example of this phenomenon is highlighted in **d** for the Bragg position marked by a circle.

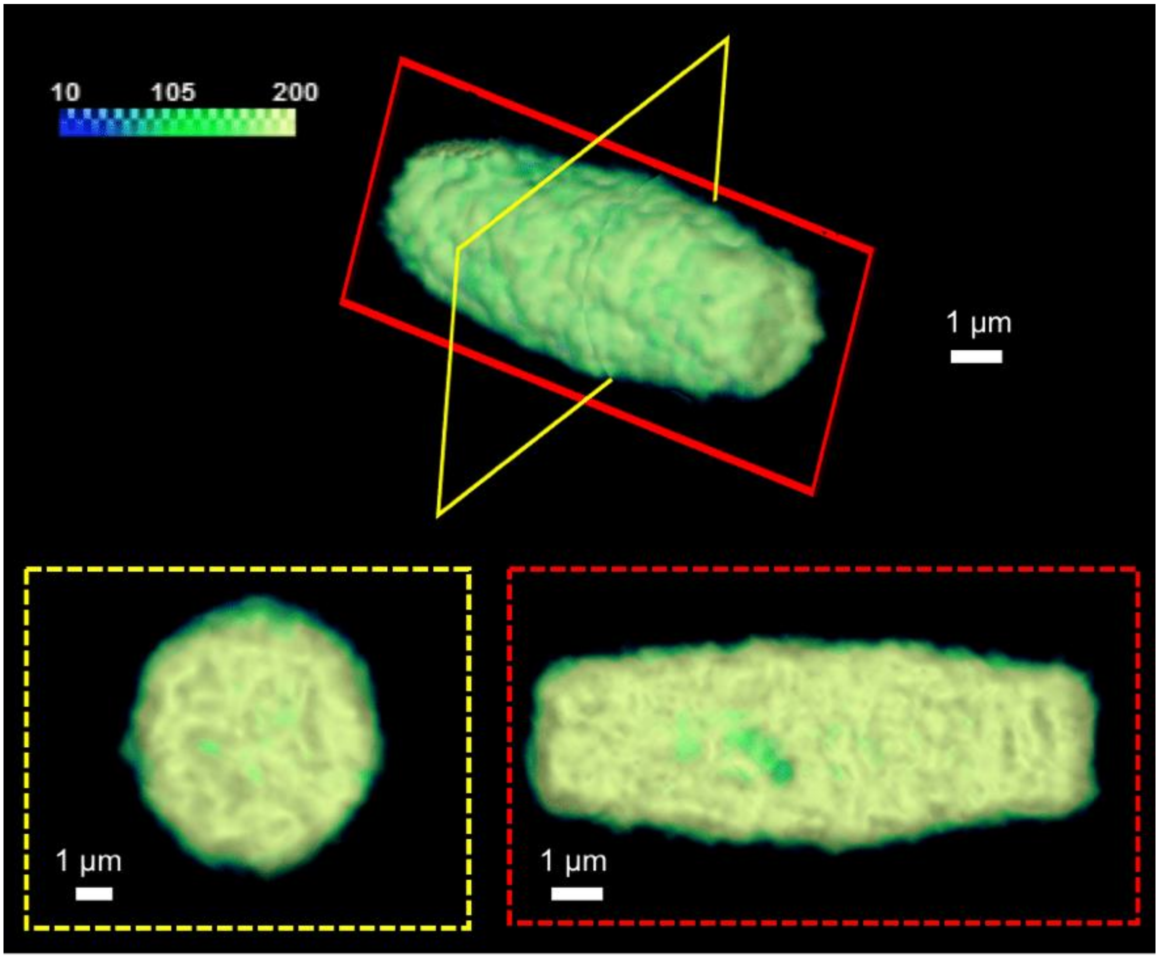


**Supplementary Fig. 17.** Micro-computed tomography full-volume rendering of **MOF-NiBr$_2$** (solvothermal method, $t$ = 48 h) (top).[4] Sections parallel (yellow frame) and perpendicular (red frame) to the base of the prismatic structure are reported in the bottom part. The color legend shows the Hounsfield unit values that are proportional to the amount of material.

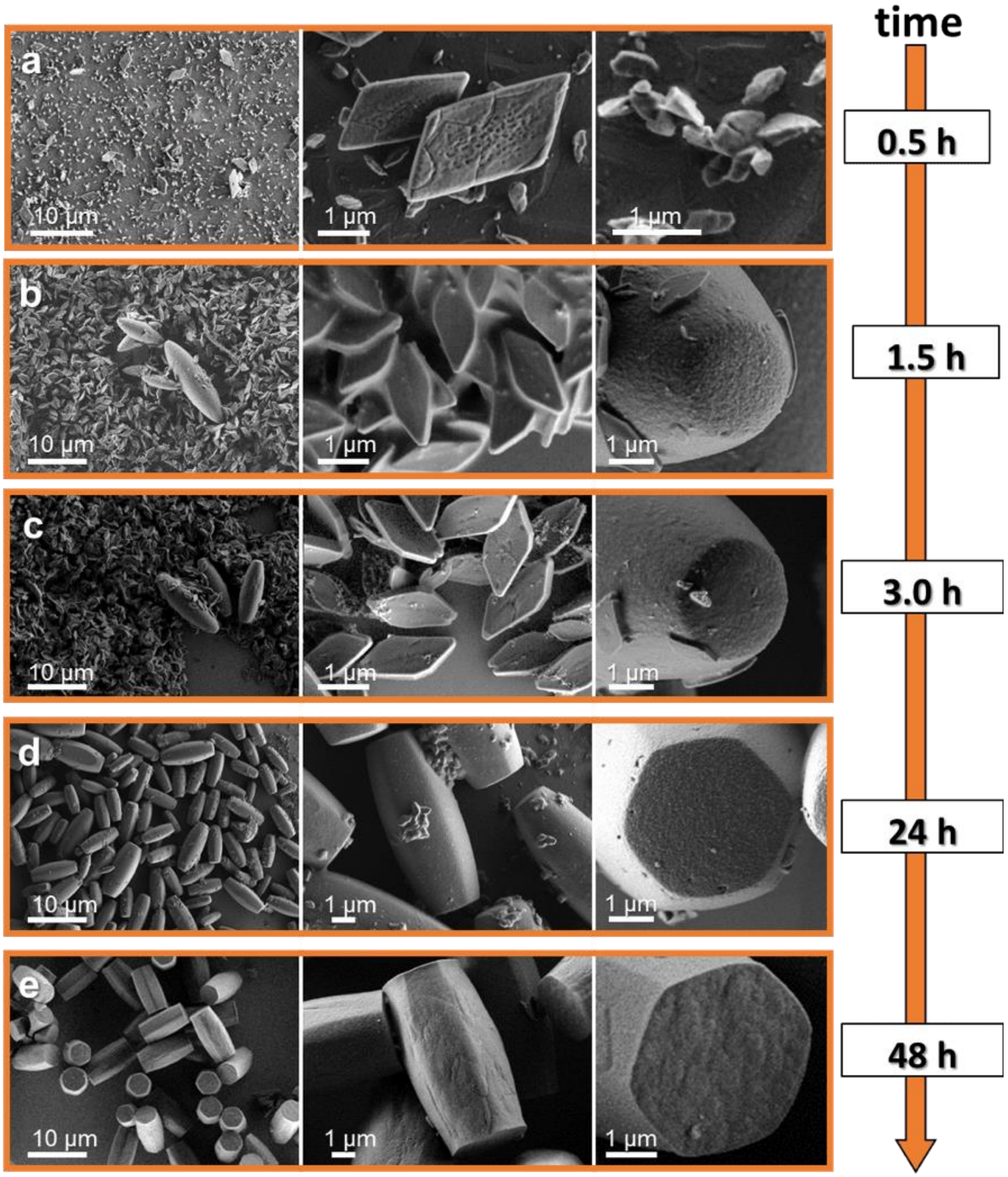


**Supplementary Fig. 18.** *Ex-situ* scanning electron microscopy (SEM) images of **MOF-NiBr$_2$** (solvothermal conditions) showing snapshots of their temporal evolution **a-e**.[4] The reaction time is denoted by the lateral arrow. Zoom-out view (left) and zoom-in micrographs of representative structures (center and right).

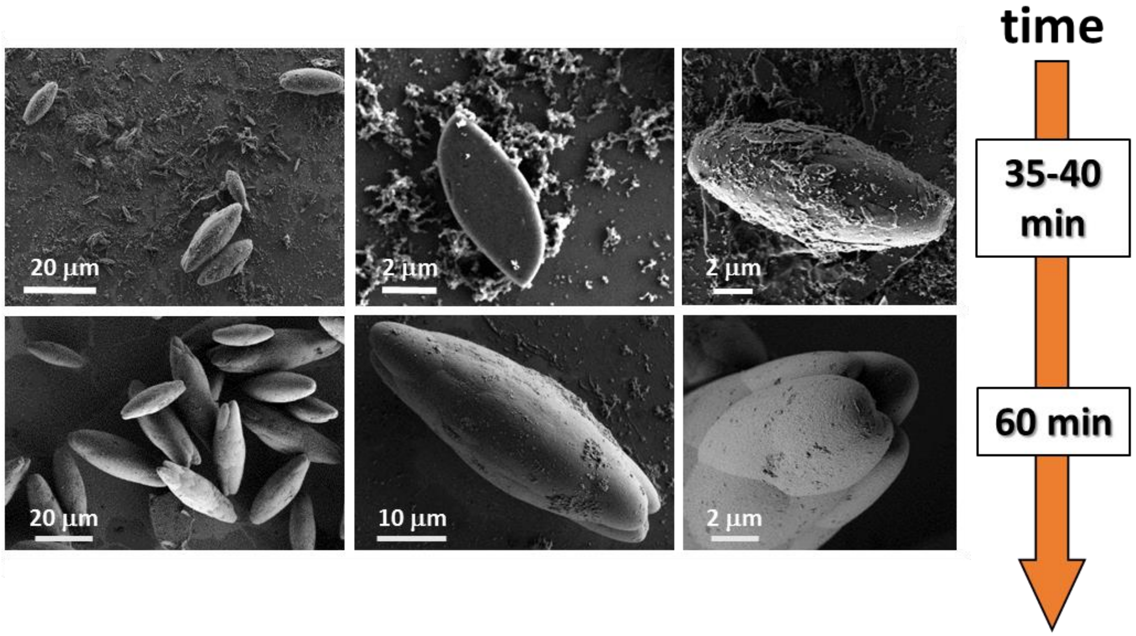


**Supplementary Fig. 19.** *Ex-situ* scanning electron microscopy (SEM) images of **MOF-NiBr$_2$** (sonochemical-solvothermal conditions) showing snapshots of their temporal evolution. The reaction time is denoted by the lateral arrow. Zoom-out view (left) and zoom-in micrographs of representative structures (center and right).

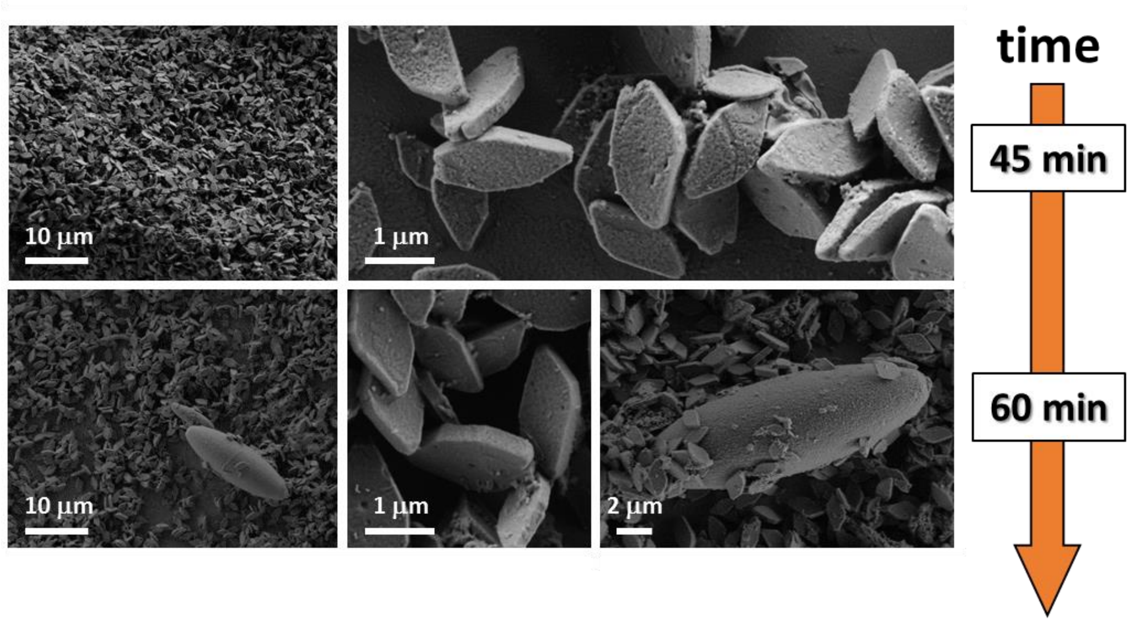


**Supplementary Fig. 20.** *Ex-situ* scanning electron microscopy (SEM) images of **MOF-NiBr$_2$** (solvothermal conditions) showing snapshots of their temporal evolution. The reaction time is denoted by the lateral arrow. Zoom-out view (left) and zoom-in micrographs of representative structures (center and right).

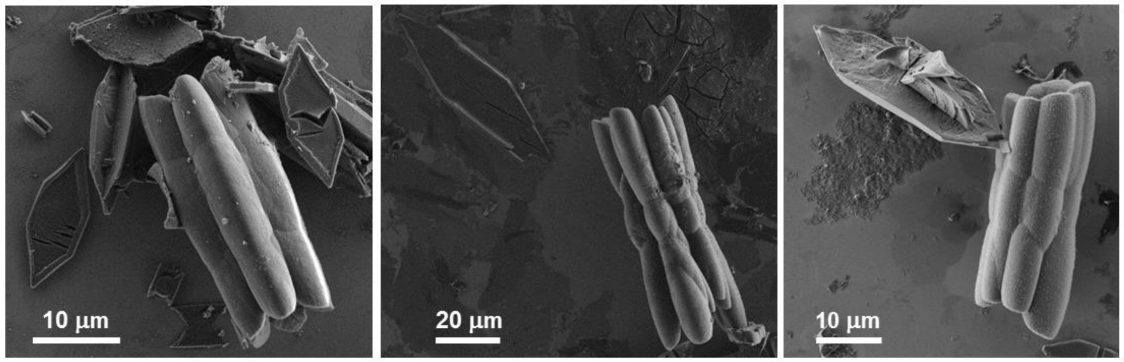


**Supplementary Fig. 21.** Scanning electron microscopy (SEM) images of **MOF-NiBr$_2$**: parallelogram crystals were isolated after solvothermal conditions (*t* = 30 min; 105°C, **TPVA**:NiBr$_2$ = 1:2). These crystals were added to the sonicated solvent (DMF and $CHCl_3$, 3:1 v/v) and further reacted under solvothermal conditions, *t* = 47.5 h, 105°C.

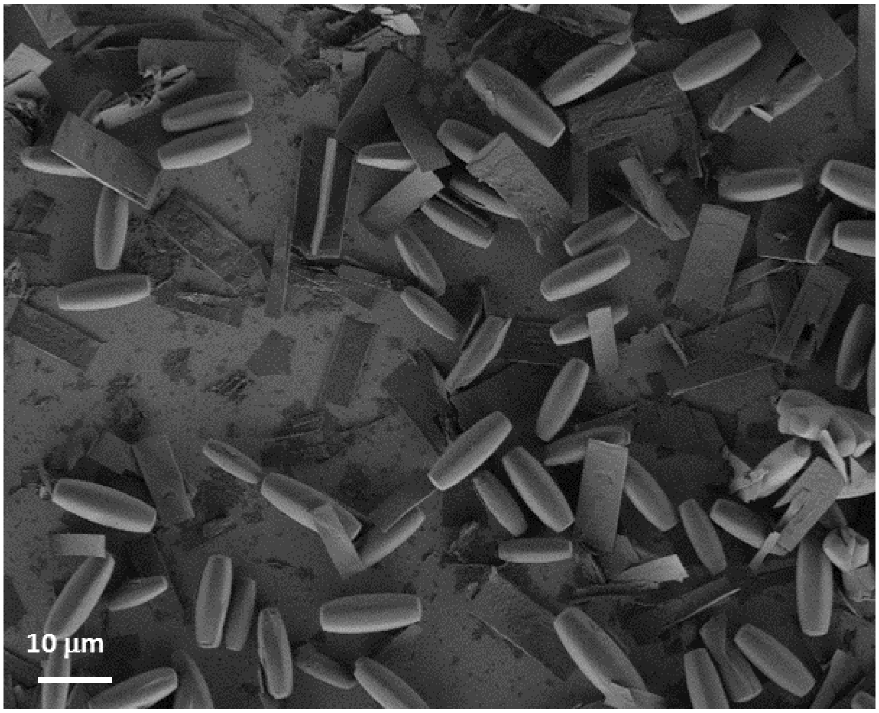


**Supplementary Fig. 22.** Scanning electron microscopy (SEM) images of **MOF-NiBr$_2$**: parallelogram crystals were isolated after solvothermal conditions (*t* = 30 min; 105°C, **TPVA**:NiBr$_2$ = 1:2). These crystals were added to the non-sonicated solvent (DMF and $CHCl_3$, 3:1 v/v) and further reacted under solvothermal conditions, *t* = 47.5 h, 105°C.

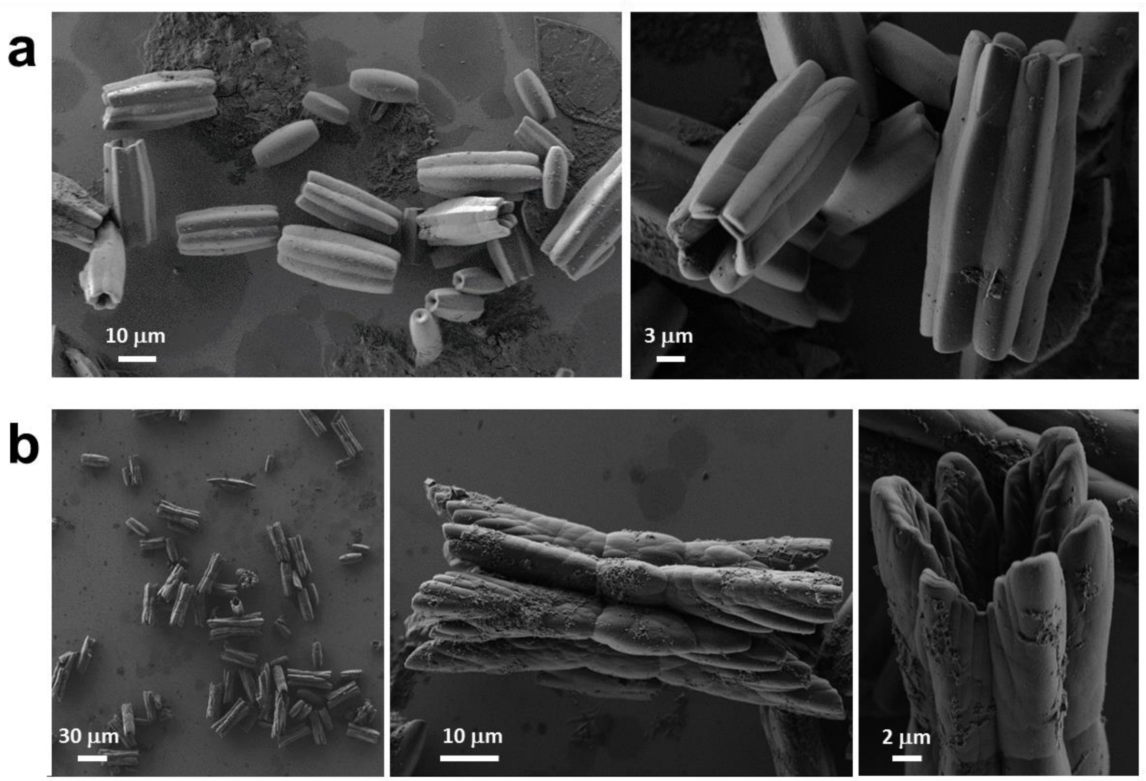


**Supplementary Fig. 23.** Scanning electron microscopy (SEM) images of representative structures of **MOF-NiBr$_2$** obtained by solvothermal conditions (105 °C, $t$ = 48 h), and **TPVA**:NiBr$_2$ (**a**) 1:1 and (**b**) 0.75:1.

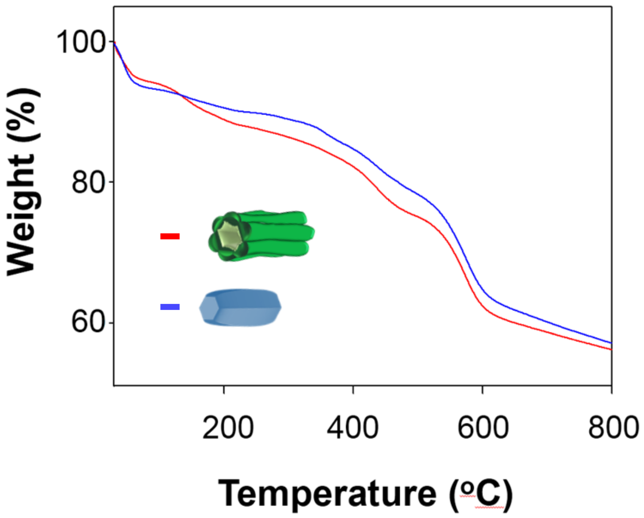


**Supplementary Fig. 24.** Thermogravimetric (TGA; under $N_2$) curves of **MOF-NiBr$_2$** obtained under (red curve) sonochemical-solvothermal conditions and (blue curve) solvothermal conditions ($t$ = 48 h, 105°C). Two initial small weight losses (4-5% and 6-7%) are observed at around 50°C and 170°C possible due to the desorption of moisture and residual solvents ($CHCl_3$, DMF). The most significant weight losses (12% and 14-16%) occurs at around 380 °C and 520°C.

**Supplementary Note 1.** The three calculated PXRD patterns from the single-crystal analysis show a high intensity peak at 6.9 degrees which corresponds to the highly diffracting, atom rich 110 plane (**Supplementary Fig. 4**, left side). The intensity of this peak is suppressed in the experimental PXRD spectra of two of the crystals (*t* = 48 h, sonication-solvothermal and solvothermal conditions) because of a distinctly preferred orientation (**Supplementary Fig. 4 a and c**, center). This orientation along the longitudinal direction is induced by their faceted morphology. The initial crystals having relatively smooth surfaces (*t* = 1.5 h. sonication-solvothermal conditions, *t* = 1.5) are less orientated on the surface, hence the presence of a strong peak. Experimental intensity variations due to preferred orientation was considered in the fits by using spherical harmonic functions[5] (**Supplementary Fig. 4**, right side).

**Supplementary Note 2**. Single-crystal X-ray data show that the crystals of **MOF-NiBr$_2$** obtained under solvothermal (*t* = 48 h)[4] and sonochemical-solvothermal (*t* = 1.5 h and *t* = 48 h) conditions are isostructural (CCDC 1996729 and CCDC 2016190, respectively). For these crystals the porosity is 37.6% of the unit cell, calculated using the contact surface employing a spherical probe with a radius of 1.2 Å. The porosity is mainly distributed in two types of continuous channels having hexagonal (⌀ ≈ 9.1 Å) and trigonal (⌀ ≈ 11.6 Å) geometries. The space group *P*622 is rare, with only 14 entries in the Cambridge Crystallographic Database. Moreover, *P*622 belongs to the 65 Sohncke space groups, thus implying the presence of chiral properties in the molecular packing. The chirality is first evident in the coordination nodes, where the pyridine moieties are arranged in a propeller fashion around the Ni atoms (**Supplementary Fig. 8)**. The overall coordination center geometry is a distorted octahedral with roughly comparable Ni–N equatorial distances (2.047 Å / 2.065, 2.021 Å / 2.037 Å and 2.100 Å / 2.08 Å for **MOF-NiBr$_2$** obtained under solvothermal conditions (*t* = 48 h)[4] and under sonochemical-solvothermal conditions (*t* = 1.5 h, *t* = 48 h), respectively.The chirality is also reflected by the ligand that is disposed helicoidally. Helicoids with the same handiness constitute the inner walls of both channels. Opposite helicoids form the molecular shell wrapping the wall of the hexagonal channels. Oxygen atoms, belonging to water molecules, are in the axial positions. The Ni–O axial distances are long: 2.470(3) Å, 2.451(7) and 2.427(6) Å for **MOF-NiBr$_2$** obtained under solvothermal conditions (*t* = 48 h)[4] and under sonochemical-solvothermal conditions (*t* = 1.5 h, 48 h), respectively. It should be noted that these extremely long Ni–O bond distances have few examples in the CCDC[11,12]. As such, alternate refinement models were also checked. The Ni atom sits on a 2–fold special position. Refinement where the O atom was replaced by Cl for an octahedral geometry with Cl atoms in the axial positions resulted in a much higher R factors and worse goodness-of-fit (GoF). **MOF-NiBr$_2$** *t* = 1.5 h (O: R = 0.0762, GoF 1.102 vs Cl: R1 = 10.16, GoF 1.402). **MOF-NiBr$_2$** *t* = 48 h (O: R1= 0.0941, GoF 1.030 vs Cl: R1 = 11.34, GoF 1.254). Also checked was the possibility of five-coordinated Ni, here a discretely disordered Cl atom was placed with 0.5 occupancy, for a total of 1 Cl atom per Ni atom. This refinement was slightly worse than the O refinement and maybe ruled out in terms of valance. While the Ni-axial distance is more consistent with Ni–Cl, all crystallographic evidence indicates an elongated Ni–O coordination as the correct choice[11,12].

| **MOF-NiBr$_2$** (sonochemical-solvothermal) | *t* = 1.5 h | *t* = 48 h |
|---|---|---|
| Measurement name | V296 | V394 |
| CCDC | 1996729 | 2016190 |
| Crystal description | Pale green barrel | Split barrel |
| Diffractometer | ESRF ID29 | Rigaku XtaLab$^{Pro}$ |
| Empirical formula | $C_{62}H_{56}N_4NiO_2$ + [solvent] | $C_{62}H_{56}N_4NiO_2$ + [solvent] |
| Formula weight (g/mol) | 947.81 | 947.81 |
| Temperature (K) | 100 | 100 |
| Wavelength (Å) | 0.700 | 1.54184 |
| Crystal system | hexagonal | hexagonal |
| Space group | *P622* | *P622* |
| a (Å) | 25.719(3) | 25.961(3) |
| b (Å) | 25.719(3) | 25.961(3) |
| c (Å) | 17.870(4) | 17.8183(16) |
| α,β, γ° | 90,90,120 | 90,90,120 |
| Volume (Å$^3$) | 10237(3) | 10400(3) |
| Z | 6 | 6 |
| Density calculated (Mg/m$^3$) | 0.922 | 0.904 |
| Absorption coefficient (mm$^{-1}$) | 0.436 | 0.673 |
| F(000) | 3000 | 2976.0 |
| Theta range for data collection (°) | 1.039 to 19.656 | 3.405 to 44.312 |
| Reflection collected (Unique) | 61522(3431) | 13070(2709) |
| $R_{int}$ | 0.0833 | 0.0630 |
| Completeness % | 98.9 | 99.1 |
| Data/restraints/parameters | 2134 /99/ 293 | 2709/276/268 |
| Goodness-of-fit on F$^2$ | 1.102 | 1.022 |
| Final R [$I>2\sigma(I)$] | R1=0.0762 wR2=0.2414 | R1=0.0941 wR2=0.2614 |
| R (all data) | R1=0. 0833 wR2=0.2527 | R1=0.1175 wR2=0.2819 |
| Largest diff. peak and hole (e$^{\cdot}$ Å$^{-3}$) | 0.165 and -0.230 | 0.406 and -0.462 |
| Flack parameter | 0.11(9) | 0.13(8) |
| Hexagonal (inner layer) and triangular channel handiness | *P* | *M* |
| Hexagonal channel (outer layer) handiness | *M* | *P* |

**Supplementary Table 1.** Crystal data and structure refinement parameters of **MOF-NiBr$_2$**, *t* = 1.5 h and *t* = 48 h, under sonochemical-solvothermal conditions. The refinement parameters clearly indicate the presence of Ni–O over Ni–Cl[11,12].

| | % C | % H | % N | % Ni | % Cl | % Br | % O |
|---|---|---|---|---|---|---|---|
| **MOF-NiBr$_2$**<br>solvothermal<br>*t* = 48 h<br>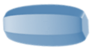 | 69.11 | 5.46 | 5.51 | 6.67 | 8.41 | 0.65 | 4.19 |
| | $[C_{62}H_{58.4}N_{4.2}Ni_{1.2}Cl_{2.6}O_{2.8}]$ | | | | | | |
| **MOF-NiBr$_2$**<br>Sonochemical-solvothermal<br>*t* = 1.5 h<br>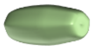 | 72.10 | 5.71 | 5.71 | 6.11 | 8.11 | 0.63 | 1.63 |
| | $[C_{62}H_{58.4}N_{4.2}Ni_{1.1}Cl_{2.2}O_{1}]$ | | | | | | |
| **MOF-NiBr$_2$**<br>Sonochemical-solvothermal<br>*t* = 48 h<br>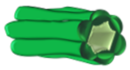 | 68.14 | 5.53 | 5.62 | 6.07 | 8.71 | 0.32 | 5.61 |
| | $[C_{62}H_{60}N_{4.4}Ni_{1.1}Cl_{2.7}O_{3.8}]$ | | | | | | |
| Calculated weight percentage | 73.10 | 5.54 | 5.50 | 5.76 | 6.96 | - | 3.14 |
| | $[C_{62}H_{56}N_{4}Ni_{1}Cl_{2}O_{2}]$ | | | | | | |

**Supplementary Table 2.** Elemental analysis data. Weight percentage of the elements constituting the bulk samples of the crystals. The theoretical weight percentage is based on a metal-to-ligand ratio of 1:1, the inclusion of two water molecules and two chloride ($Cl^-$) anions. The presence of $Cl^-$ was observed in metal-organic crystals[4,6,7] and attributed to the metal-mediated chloroform decomposition under solvothermal conditions.[8] Traces of $NiCl_2$ might be present in the samples. The estimated molecular formulas are shown between the square brackets.

## Supplementary References


1. V. Mišík, P. Riesz, Peroxyl radical formation in aqueous solutions of *N,N*-dimethylformamide, *N*-methylformamide, and dimethylsulfoxide by ultrasound: Implications for sonosensitized cell killing, *Free Radical Bio Med* **20**, 129-138 (1996).

2. V. Mišík, P. Riesz, Free radical formation by ultrasound in organic liquids: A spin trapping and EPR study, *J. Phys. Chem.* **98**, 1634-1640 (1994).

3. C. L. Øpstad, T.-B. Melø, H.-R. Sliwka, V. Partali, Formation of DMSO and DMF radicals with minute amounts of base, *Tetrahedron* **65**, 7616-7619 (2009).

4. Wen, Q., Tenenholtz, S., Shimon, L. J. W., Bar-Elli, O., Beck, L., Houben, L., Cohen, S. R. , Feldman, Y., Oron, D., Lahav, M., van der Boom, M. E., Chiral and SHG-active metal-organic frameworks in solution and on surfaces: Uniformity, morphology control, oriented growth and post-assembly functionalization, *J. Am. Chem. Soc.* **142**, 14210-14221 (2020), CCDC 1996729.

5. Järvinen, M. Application of symmetrized harmonics expansion to correction of the preferred orientation effect. *J. Appl. Cryst.* **26**, 525-531 (1993).

6. di Gregorio, M.C., Shimon, L.J.W., Brumfeld, V. et al. Emergence of chirality and structural complexity in single crystals at the molecular and morphological levels. *Nat Commun* **11,** 380 (2020).

7. di Gregorio, M.C., Ranjan, P., Houben, L. et al. Metal-coordination-induced fusion creates hollow crystalline molecular superstructures *J. Am. Chem. Soc.* **140**, 29, 9132–9139 (2018).

8. Zhu, L. & Bozzelli, J. W. Kinetics and mechanism for the thermal chlorination of chloroform in the gas phase: Inclusion of HCl elimination from $CHCl_3$ *Int. J. Chem. Kinet.* **35**, 647-660 (2003)

9. MiŠík, V. and Riesz, P. Free radical formation by ultrasound in organic liquids: A spin trapping and EPR study. *J. Phys. Chem.* **98**, 1634-1640 (1994).

10. Riesz, P., Berdahl, D. and C. Christman, L. Free radical generation by ultrasound in aqueous and nonaqueous solutions. *Environ. Health Perspect.* **64**, 233-252 (1985).

11. Yilmaz, A.; Camurlu, P.; Yıldırım, L. T. et al. Synthesis, crystal structure and spectral analysis of diaquatetrakis(*N*-methylimidazole)Ni(II)(2,4,6-tribromophenol). *Cryst. Res. Technol.* **41**, 829-835 (2006).

12. Sarkar, S.; Mukherjee, T. Sen, S. et al. Copper(II) complex of *in situ* formed 5-(2-pyridyl)-1,3,4-triazole through C–S bond cleavage in 1,2-bis(2-pyridylmethylthio)-bis-ethylsulphide: Synthesis, structural characterization and DNA binding study. *J. Mol. Struct.* **980**, 117-123 (2010).
