# Supplementary material for: Molecular cannibalism: Sacrificial materials as precursors for hollow and multidomain single crystals
Source: Nat Commun. 2021 Feb 11;12:957. doi: 10.1038/s41467-021-21076-9 (PMC7878748; doi:10.1038/s41467-021-21076-9)
Supplement: Supplementary file 2 — Description of Additional Supplementary Files [file 41467_2021_21076_MOESM2_ESM.pdf]

Description of Additional Supplementary Files

File Name: Supplementary Movie 1
Description: Micro-CT volume rendering of **MOF-NiBr$_2$** (sonochemical-solvothermal conditions, t = 48 h). Full volume reconstruction and progressive slicing along the cavity axis are shown. Snapshots are reported in Figs. 2e and 4f.

File Name: Supplementary Movie 2.
Description: Micro-CT volume rendering of **MOF-NiBr$_2$** (sonochemical-solvothermal conditions, t = 1.5 h). Progressive slicing along the longitudinal direction of the structure. Snapshots are reported in Figs. 4a.
